# Characteristics of respiratory viruses’ circulation through a six-year period (2016–2022) in a pediatric population in Normandy, France, and the impact of COVID-19 pandemic

**DOI:** 10.1128/spectrum.01867-23

**Published:** 2023-10-26

**Authors:** J. Dina, A. Moisan, P. Thibon, C. Creveuil, J. Adnet, A. Vabret, J. Brouard, J. C. Plantier

**Affiliations:** 1 INSERM U1311, Dynamicure, UNICAEN, UNIROUEN, Virology Department, CHU Caen, Paris, France; 2 INSERM U1311, Dynamicure, UNIROUEN, UNICAEN, Virology Department, CHU de Rouen, France; 3 Centre d’appui pour la Prévention des Infections Associées aux Soins, CPias Normandie, CHU de Caen, France; 4 Statistic Department, CHU de Caen, France; 5 INSERM U1311, Dynamicure, UNICAEN, UNIROUEN, Pediatrics Department, CHU Caen, France; University Hospital of Reims and Faculty of Medicine, University of Reims Champagne Ardenne, Reims, France

**Keywords:** respiratory viruses, epidemiology, RSV, influenza virus, COVID-19 pandemic

## Abstract

The impact of COVID-19 on the circulation of respiratory viruses has highlighted the need for ongoing surveillance and preparedness for emerging infectious diseases. The aim of this study was to provide information on the dynamics of respiratory viruses in pediatric populations to inform public health policies and strategies for the prevention and control of respiratory infections. This study analyzed data on the detection of respiratory viruses in pediatric populations from 2016 to 2022. The weekly number of tests and positive results for respiratory viruses were collected and analyzed from the two major university hospitals, Caen and Rouen. Different assays, including multiplex panels and rapid detection kits, were used for testing. The study analyzed the epidemiological trends of respiratory viruses in a French region during the COVID-19 pandemic. The data set included 37,969 tests, with a significant increase in the number of tests in 2020–2021 and 2021–2022. The rate of positive tests fell below 50% in 2021–2022. Enteroviruses (EV)/rhinoviruses (RV) and respiratory syncytial viruses (RSV) were the most frequently identified viruses, except in 2021–2022, when Flu ranked second. The Flu epidemic appeared slightly earlier in 2021–2022 and lasted for 25 weeks, with a doubling time of 4.03. The RSV epidemic occurred earlier in 2021–2022 and had similar dynamics to pre-pandemic years. EV/RV continued to circulate throughout the lockdowns, while low numbers of other viruses were detected. Our results emphasize the importance of continuing active surveillance of respiratory virus circulation and the need for new recommendations for respiratory virus detection in pediatric patients.

**IMPORTANCE:**

The report highlights an epidemiological change in the circulation of respiratory viruses in pediatric populations due to strategies adopted against COVID-19 pandemic. COVID-19 has resulted in a significant increase in requests for multiplex respiratory research to identify the virus responsible for the symptoms. The diagnostic needs have increased, and the number of samples analyzed in 2021–2022 is equal to the samples analyzed over the four epidemic periods preceding the pandemic. The report suggests the importance of active surveillance of respiratory viruses' circulation and new recommendations for respiratory virus detection in pediatric patients.

## INTRODUCTION

Respiratory infections are a significant global health issue, with viruses being responsible for over 80% of all respiratory tract infections (1). In children, the majority of respiratory viral infections are self-limited. Nevertheless, viruses are responsible for one-quarter of hospitalizations and up to 60% of visits to the general practitioner (2, 3). Common respiratory viral pathogens include adenoviruses, enteroviruses (EV), human coronaviruses, human metapneumoviruses, rhinoviruses (RV), influenzaviruses, parainfluenza viruses (PIV), and respiratory syncytial viruses (RSV). RSV are known as the most important cause of respiratory tract infections leading to hospitalization among infants (4, 5). RV cause the majority of colds and exacerbations of asthma (6, 7). Influenza are the most common cause of pneumonia-related deaths in high-resource countries (8).

The major impact of respiratory infections on public health, the organization of care, and the management of patients and their diagnosis explain the need for knowledge on the epidemiology of the different viruses involved.

Outbreaks of respiratory viruses like RSV, human metapneumoviruses (hMPV), seasonal coronaviruses, and influenzaviruses occur each winter season in temperate climates, whereas lower activity is detected during the summer months. Other human respiratory viruses, such as PIV-1, PIV-2, PIV-3, and PIV-4 and RV, show the highest activity during the spring or fall season in temperate regions (9).

The disruption of these known situations produced by the emergence of SARS-CoV-2 has generated growing interest in the impact of the COVID-19 pandemic on the circulation of other respiratory viruses. Some authors have described the changes in the seasonal circulation of respiratory viruses other than SARS-CoV-2, in particular, influenza and RSV, evoking their immediate disappearance after the application of sanitary measures at the international level and their increase in subsequent years (10 – 14).

The aim of this study was to describe the changes in respiratory virus circulation profile in a pediatric population between 2016 and 2022, focusing on the impact of SARS-CoV-2 emergence and the lifting of sanitary measures.

## MATERIALS AND METHODS

Data on the detection of respiratory viruses, in pediatric populations under the age of 16 years, were analyzed during the period 2016–2022. In this study, we considered “epidemic years,” i.e., from 1 September of the current year to 31 August of the following year, corresponding to seasonality in the Normandy region of France.

The two major public hospitals, the University Hospital of Caen (1,433 beds) and the University Hospital of Rouen (2,450 beds), were included in this study. The weekly number of tests performed and the positive results for each respiratory virus were collected and analyzed.

Samples from infants with respiratory symptoms, sent for routine diagnostic purposes, were tested using different assays depending on the epidemic period, the need for emergency diagnosis, and the medical prescription. The panel of viruses tested and the assays used for their detection are presented in Table S1.

The different workflows were performed as recommended by the manufacturers. Multiplex panel allows the identification of influenzaviruses, RSV, adenoviruses, EV, PIV-1, PIV-2, PIV-3, PIV-4, metapneumoviruses, bocaviruses, RV, and seasonal coronaviruses NL63/HKU1/229E/OC43.

### Lockdown periods

Three lockdown periods have been implemented in France including control measures, such as school and business closures, travel restrictions, social distancing, and mask wearing to limit the circulation of SARS-CoV-2. The first period, from 17 March 2020 to 11 May 2020, was followed by a reopening period with gradually lightened rules until the end of June. The second period due to a new wave of COVID-19 was declared from 30 October 2020 until 15 December 2020. The third one was from 3 April 2021 to 3 May 2021.

### Statistical analysis

For each season, we calculated (i) the percentage of positive tests for all viruses (number of positive tests/total number of tests) and for each type of virus (number of positive tests for each virus/total number of tests), (ii) the distribution of virus types among the positive tests (number of positive tests for each virus/total number of positive tests), and (iii) the weekly mean and standard deviation of test positivity (number of positive tests for each virus/total number of tests) for the autumn–winter period (October to April).

For influenza and RSV, several parameters were used to describe the six epidemic seasons. (i) For each virus, the mean of the weekly number of viruses identified during the period of low circulation of respiratory viruses, from May to September, plus two standard deviations (2SD) was used as a threshold to determine the start of the epidemic. Crossing the threshold for two consecutive weeks defined the beginning of the epidemic. (ii) The epidemic peak was the week with the highest percentage of positive specimens between the beginning and end of the epidemic. (iii) The duration of the epidemic was the number of weeks between the start and the end of the epidemic (number of positive samples below the threshold for two consecutive weeks. (iv) The doubling time was calculated by fitting an exponential curve to the weekly proportions of positive tests for the first 4 weeks of each epidemic season.

The non-parametric Kruskal-Wallis test was used to compare the weekly mean of positive tests between November and April of each season, and the chi-square test was used to compare values at the peak between epidemic seasons.

Data were compiled using Excel 2018 (Microsoft) and analyzed using R version 4.0. A value of *P* < 0.05 was considered significant.

Institutional review board was not required because the study constitutes a surveillance/outbreak investigation that was stripped of all individual information.

## RESULTS

**Fig 1:**
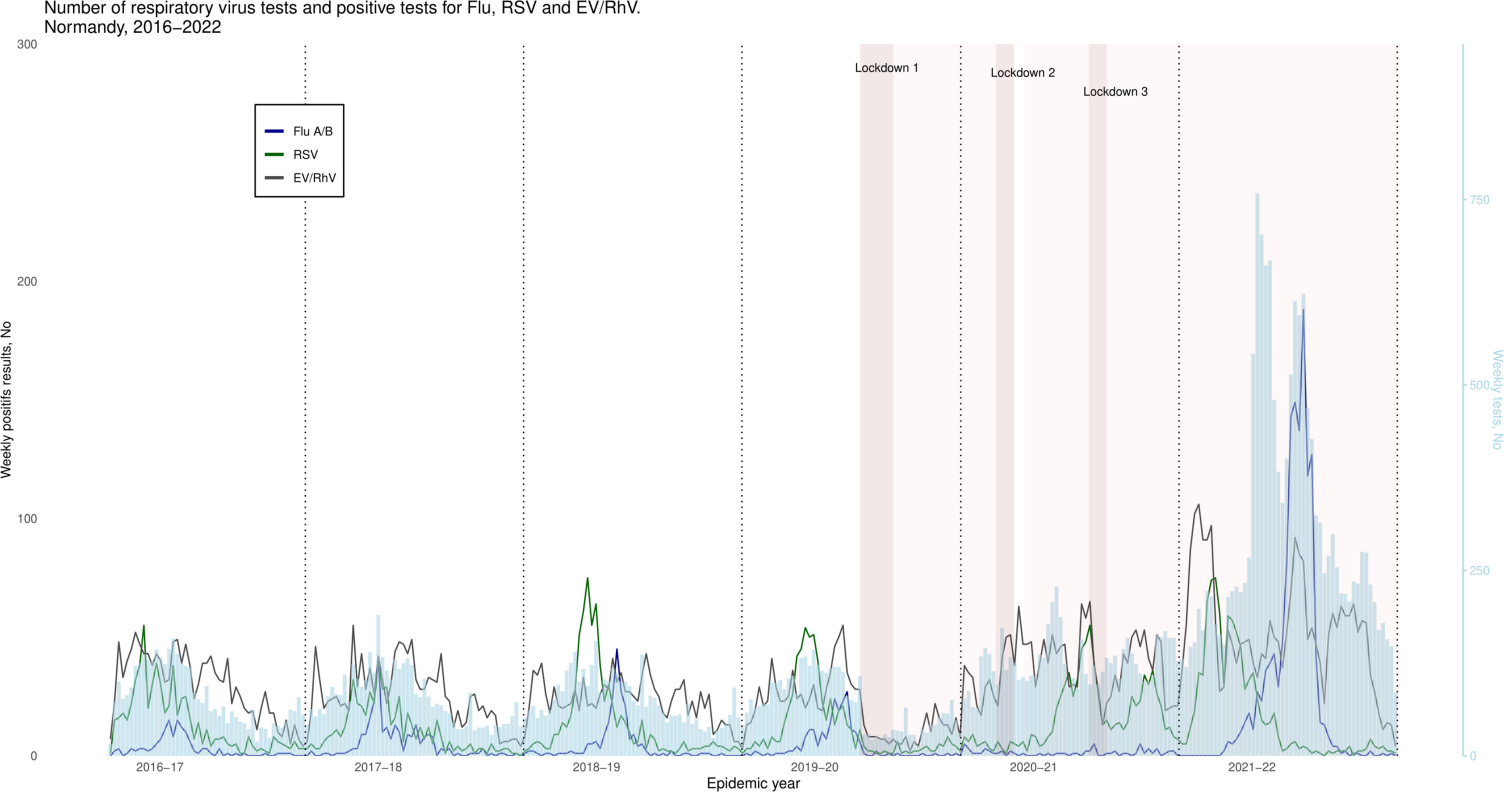
Weekly numbers of respiratory virus tests and positive tests for influenza, respiratory syncytial viruses, and enterovirus/rhinovirus. Normandy, France, 2017–2022. Only viruses accounting for more than 10% of positive results were represented: Flu A/B: influenza viruses A and B, RSV: respiratory syncytial viruses A and B, EV/RV: enterovirus/rhinovirus.

The data set comprised 37,969 tests, with a marked increase in the number of tests in 2020–2021 (17.4% of the total) and especially in 2021–2022 (42.0%). During this last season, the rate of positive tests fell below 50%, whereas in other seasons, it was around 70%. In each year, EV/RV were the most frequently identified viruses (40% of positive tests), and RSV were the second most frequently identified viruses (20%), except in 2021–2022 when influenza viruses came second (19.7% of positive tests versus 12.9% for RSV) (Table 1; Fig. 1; Table S1; Fig. S1 and S2). The other viruses studied represented less than 10% of all the viruses detected each year.

**TABLE 1:** Total samples analyzed and infections detected in Caen and Rouen University Hospitals during the six studied seasons^*d*^

Seasons	Total samples analyzed N1	Total positive N2 (%)^*a*^	Flu *N*+ (%)^*b*^	RSV *N*+ (%)^*b*^	EV/RV *N*+ (%)^*b*^	PIV *N*+ (%)^*b*^	CoV *N*+ (%)^*b*^	hMPV *N*+ (%)^*b*^	BoV *N*+ (%)^*b*^	ADV *N*+ (%)^*b*^
2016–2017	3737	3143 (84.1)	132 (4.2)	627 (19.9)	1388 (44.2)	182 (5.8)	200 (6.4)	198 (6.3)	225 (7.2)	191 (6.1)
2017–2018	4053	3061 (75.5)	245 (8.0)	532 (17.4)	1342 (43.8)	190 (6.2)	204 (6.7)	178 (5.8)	189 (6.2)	181 (5.9)
2018–2019	3936	3017 (76.7)	213 (7.1)	695 (23.0)	1241 (41.1)	170 (5.6)	158 (5.3)	184 (6.1)	174 (5.8)	182 (6.0)
2019–2020	3680	2478 (67.3)	212 (8.6)	607 (24.5)	1018 (41.1)	111 (4.5)	142 (5.7)	151 (6.1)	129 (5.2)	108 (4.4)
2020–2021	6613	4572 (69.1)	65 (1.4%)	847 (18.5)	1990 (43.5)	426 (9.3)	300 (6.6)	259 (5.7)	321 (7.0)	364 (8.0)
1st lockdown^*c*^	219	100 (45.7)	4 (4.0)	0 (0)	63 (63.0)	3 (3.0)	6 (6.0)	2 (2.0)	15 (15.0)	7 (7.0)
2nd lockdown^*c*^	850	458 (53.9)	7 (1.5)	0 (0)	311 (67.9)	9 (2.0)	9 (2.0)	21 (4.6)	13 (2.8)	88 (19.2)
3rd lockdown^*c*^	559	413 (73.9)	8 (1.9)	0 (0)	166 (40.2)	20 (4.8)	84 (20.3)	43 (10.4)	51 (12.3)	41 (9.9)
2021–2022	15950	6874 (43.1)	1355 (19.7)	888 (12.9)	2695 (39.2)	434 (6.3)	310 (4.5)	309 (4.5)	314 (4.6)	569 (8.3)

^*a*^ % reported to total samples analyzed (N2/N1).

^*b*^ % reported to total positive samples (N+/N2).

^*c*^ 1st lockdown: 2020/03/17-2020/05/11, 2nd lockdown: 2020/10/30-2020/12/15, 3rd lockdown: 2021/04/03-2021/05/03.

^*d*^ N+: number of positive tests, ADV: adenoviruses,BoV: bocavirus, Cov: coronaviruses, EV/RV: enteroviruses/rhinoviruses, Flu: influenza viruses, hMPV: human metapneumovirus, PIV: parainfluenza viruses, RSV: respiratory syncytial virus, and POS: positive.

**Fig 2:**
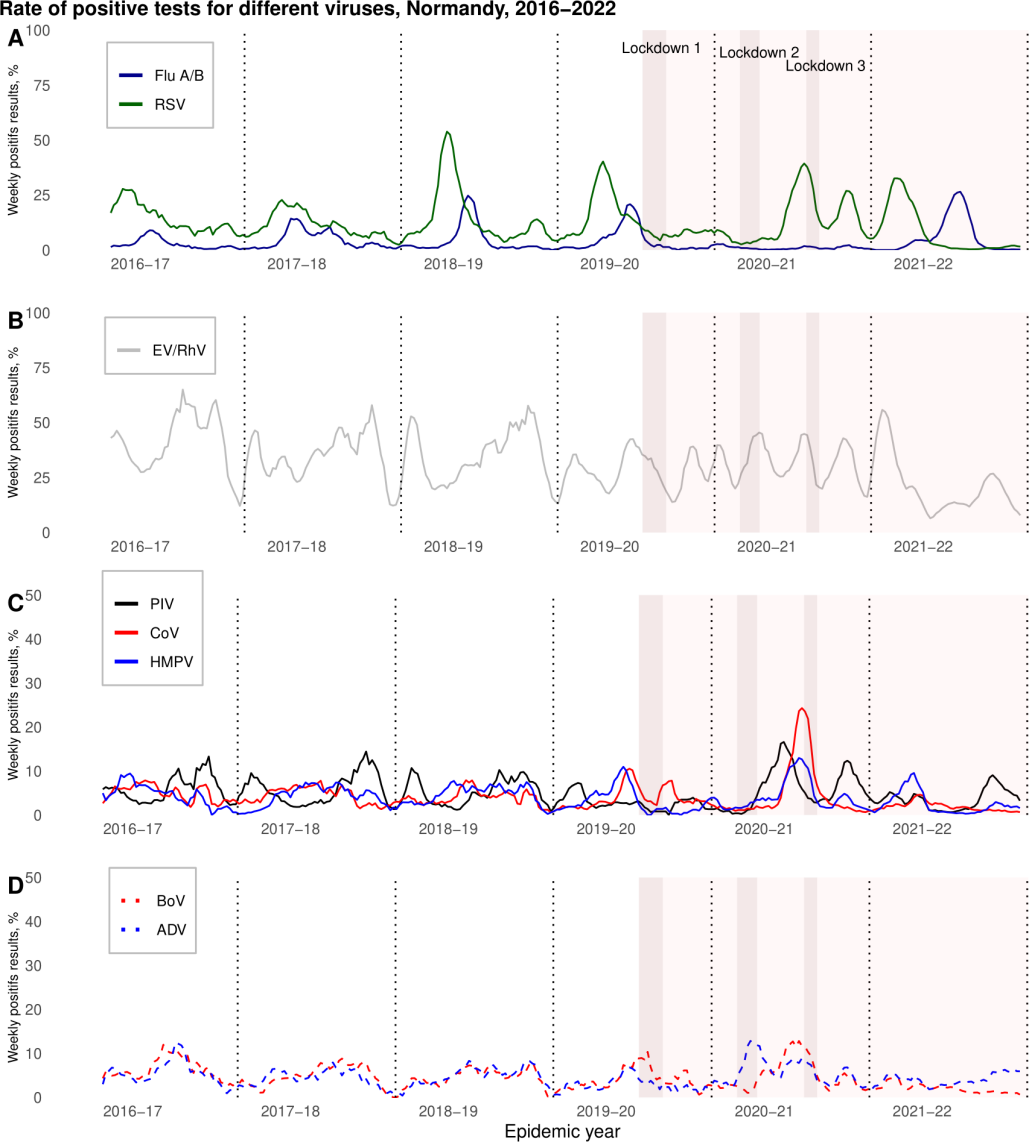
Weekly proportion of positive respiratory virus tests. Normandy, France, 2017–2022. The curves correspond to the moving averages of order 5 of the number of positive tests divided by the total number of test (all viruses combined). Vertical dotted line: 1 September (week 36) of each season. Flu A/B: influenza viruses A and B, RSV: respiratory syncytial viruses A and B, EV/RV: enterovirus/rhinovirus, PIV: parainfluenza viruses 1, 2, 3, and 4, CoV: coronaviruses NL63, HKU1, 229E, and OC43.

**Fig 3:**
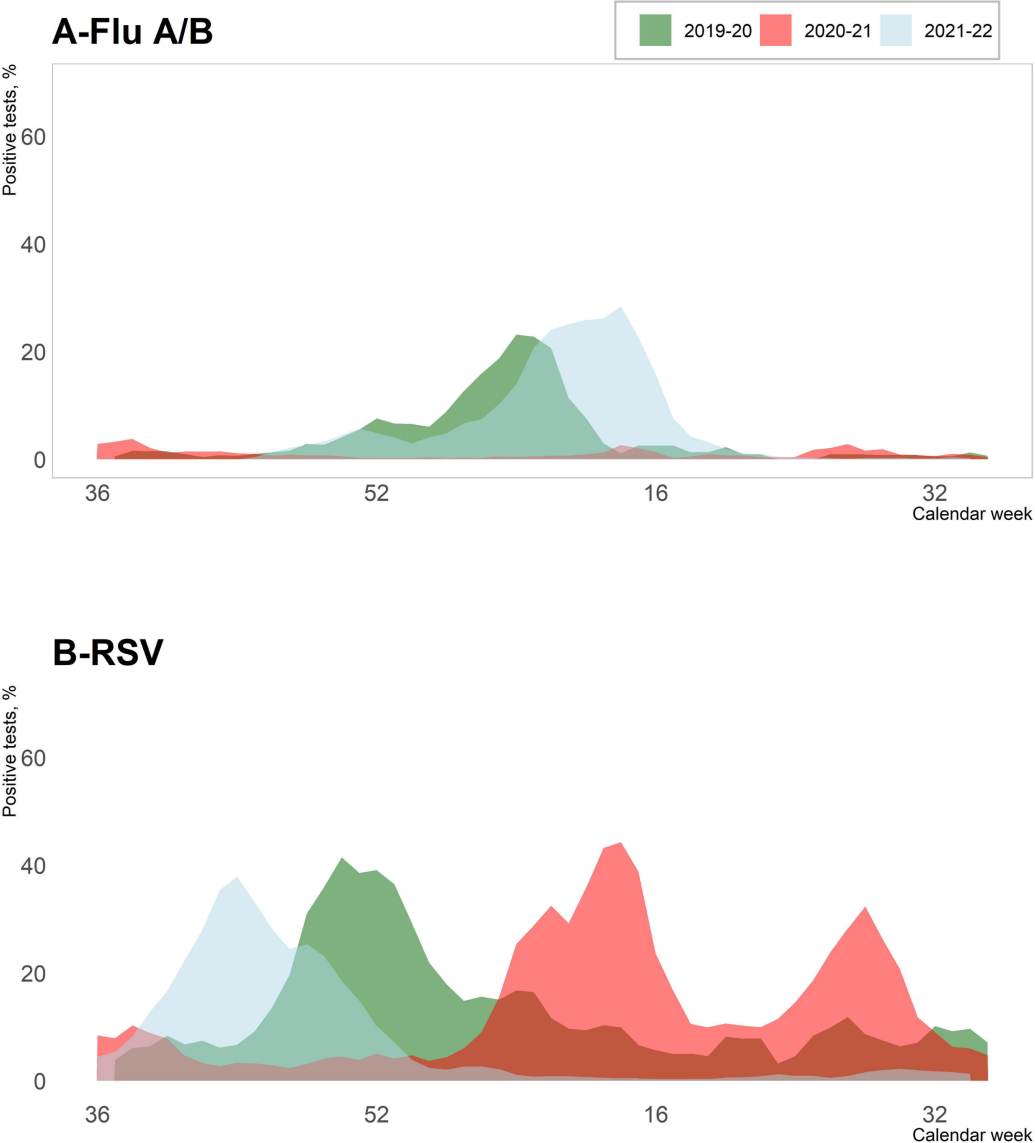
Influenza and respiratory syncytial viruses: comparison of proportion of positive virus respiratory tests for seasons 2019–2020, 2020–2021, and 2021–2022. Normandy, France. Data presented are number of positive tests divided by the total number of tests (all viruses combined). Flu A/B: influenza viruses A and B, RSV: respiratory syncytial viruses A and B.

The Flu epidemic onset week was between W49 and W52 for the pre-pandemic period. In the 2020–2021 season, no influenza epidemic was identified, with only 65 positive cases detected in our region. In the 2021–2022 season, the Flu epidemic appeared slightly earlier (W47) and lasted 25 weeks (compared to 14 weeks in 2019–2020) with a peak at W13 later than in previous years and a doubling time of 4.03, higher than in previous years (Table 2; Fig. 2 and 3).

**TABLE 2:** Number of samples and characteristics of annual epidemics for eight viruses. Normandy, France^*g*^

Season Influenza A and B	2016–17	2017–18	2018–19	2019–20	2020–21	2021–22
Positive tests^*a*^ (%), mean (SD)	3.7 (3.2)	6.8 (5.5)	6.3 (9.4)	6.7 (7.9)	0.8 (0.9)	9.2 (10.1)^*f*^
Epidemic:						
Start week	2016-W52	2017-W49	2018-W51	2019-W51	-^*b*^	2021-W47
Week of the peak	2017-W05	2018-W01	2019-W06	2020-W09	-	2022-W13
Value at peak, n/N (%)	15/137 (10.9)	40/190 (21.1)	45/111 (40.5)	27/81 (33.3)	-	188/623 (30.2)^*f*^
Duration (weeks)	9	10	10	14	-	25
Doubling time (weeks)	1.83	2.77	<0	<0	-	4.03
Respiratory syncytial virus						
Positive tests^*a*^ (%), mean (SD)	17.7 (7.9)	14.9 (6.6)	19.3 (18.3)	17.2 (12.6)	13.9 (15.1)	11.7 (13.1)^*d*^
Epidemic:						
Start week Week of the peak	2016-W46 2016-W49	2017-W46 2017-W46	2018-W48 2018-W51	2019-W47 2019-W49	2021-W06 2021-W14	2021-W39 2021-W44
Peak week value, n/N (%)	55/137 (40.1)	20/73 (27.4)	75/94 (79.8)	42/84 (50.0)	55/96 (57.3)	75/77^*e*^ (42.4)
Duration (weeks)	14	10	10	14	11	16
Doubling time (weeks)	4.09	<0	1.62	2.83	1.38	2.56
Enteroviruses/rhinoviruses						
Positive tests^*a*^ (%), mean (SD)	41.5 (14.9)	33.8 (9.9)	30.9 (11.8)	30.0 (9.1)	32.6 (13.3)	18.9 (13.2)^*f*^
Parainfluenza viruses						
Positive tests ^*a*^ (%), mean (SD)	5.2 (3.6)	4.3 (3.0)	4.6 (3.9)	3.0 (2.7)	6.3 (6.2)	2.5 (2.2)^*d*^
Coronaviruses						
Positive tests^*a*^ (%), mean (SD)	5.6 (2.5)	4.5 (2.9)	4.2 (2.7)	3.9 (3.7)	7.1 (10.0)	2.4 (1.5)^*e*^
Human metapneumovirus						
Positive tests^*a*^ (%), mean (SD)	6.0 (3.4)	4.9 (3.2)	5.1 (3.0)	4.2 (3.8)	4.4 (4.9)	3.1 (3.6)^*d*^
Bocavirus						
Positive tests^*a*^ (%), mean (SD)	6.9 (3.8)	4.8 (2.5)	5.0 (3.0)	4.8 (4.4)	6.5 (5.1)	2.6 (1.8)^*e*^
Adenoviruses						
Positive tests^*a*^ (%), mean (SD)	6.2 (4.6)	5.0 (3.0)	5.2 (2.7)	3.7 (3.0)	7.3 (4.3)	3.6 (1.6)^*d*^

^*a*^ Weekly rates (number of positive tests divided by total number of tests—all viruses combined), from October to April.

^*b*^ No epidemic identified.

^*c*^ Non-significant difference.

^*d*^ *P*<0.01.

^*e*^ *P*<0.001.

^*f*^ *P*<0.0001.

^*g*^ No testing possible.

The RSV epidemic appeared as early as W06 with a peak in W14 (early April) and a second peak in W27 (early July) in 2020–2021, whereas it had appeared between W46 and W48 (November) with a single peak between W49 and W51 (December) in previous years. In 2021–2022, the RSV epidemic occurred in W39 (late September) and had similar dynamics to the pre-pandemic years (Table 2; Fig. 2 and 3). Figure 3 highlights the epidemic particularities of the influenza viruses and RSV around the period of emergence of SARS-CoV-2. For RSV, a double epidemic peak was observed, and for influenza viruses, the absence of circulation was observed. EV/RV circulated all over the year during the six analyzed seasons, with no apparent decrease in relation to lockdown. In the season 2020–2021, the peaks for PIV, coronaviruses, hMPV, bocaviruses, and adenoviruses were the highest for the whole study period (15.9% in W05, 39.6% in W14, 19.8% in W14, 19.0% in W10, and 18.6% in W50, respectively), and weekly positive rates from October to April were also the highest during that season. For these five virus types, with the exception of hMPV, the lowest positivity rates from April to October were observed in the 2021–2022 season (Table 2; Fig. 2; Table S1).

Low numbers of viruses were detected throughout the two months of the first lockdown. Thereafter, EV/RV recovered very rapidly and predominated during the second and third lockdowns (>60% of all viruses detected). The other respiratory viruses began circulating again later, after the second containment period (Table 1; Fig. 2).

## DISCUSSION

This report describes the epidemiology of respiratory viruses in hospitalized children under 16 years old in two University Hospitals of Normandy region, France, spanning a period of 6 years (2016–2022). The significance of this study comes from the fact that respiratory infections are causing a serious public health concern particularly among children. During the 3 years preceding the SARS-CoV-2 emergence (2016–2017, 2017–2018, and 2018–2019), EV/RV, RSV, and influenzaviruses were most commonly detected. Before COVID-19 pandemic, the pediatric population was exposed to a variety of viruses with similar seasonal patterns, each virus had a similar circulation profile from one year to the next (15). According to other epidemiological studies in temperate regions, before COVID-19 pandemic, it was observed that a majority of respiratory viruses exhibited seasonal variations. Specifically, the influenza viruses, human coronaviruses, and human RSV tended to have their highest incidence during the winter months (16 – 19). On the other hand, adenoviruses, human bocaviruses, hMPV, and RV were detectable year-round (18, 20, 21). Some EV also displayed an increased detection frequency and case numbers during the summer months (22, 23). Additionally, PIV demonstrated a type-specific pattern of seasonal circulation (24).

The period from September 2019 to September 2020 is the epidemic period that began with a classic circulation of respiratory viruses followed in 2020–2021 by a sudden change as soon as non-pharmaceutical interventions (NPIs) such as mask use, social distancing, or mobility restriction were put in place at the global level. During the COVID-19 pandemic, the circulation of viruses was abruptly stopped after the implementation of the first lockdown measures. The 2020–2021 season has also been impacted, and a modified pattern of respiratory viruses’ circulation was noted. Circulation intensified during the second post-pandemic season for all respiratory viruses with significant differences in circulation periods and durations compared to seasons preceding the emergence of SARS-CoV-2.

The identification of the pre-peak periods and the epidemic peak are usually defined a posteriori. Epidemic periods are defined each year by the health authorities, as the circulation of viruses evolves, on the basis of multiple criteria including the increase in the number of positive samples reported by the laboratories, the number of visits to the emergency room for respiratory symptoms, and the increase in the number of respiratory infections seen by sentinel doctors. To describe each season, we used several parameters including calculating the threshold for defining the beginning and the end of the epidemic, identifying the epidemic peak, and calculating the doubling time. Using this methodology, it becomes possible to compare data from multiple epidemic seasons in an understandable way.

There are two events that emerge from this analysis. The first one is the more intensive circulation of EV compared to other respiratory viruses after the application of the various restrictions. The second one is the disappearance of influenza viruses for a long period after the first lockdown.

The peak of influenza occurred from December to March during the four epidemic periods before SARS-CoV-2 pandemic with a pre-peak that spreads out from weeks 49 to 52. During the 2020–2021 epidemic period, from September 2020 to August 2021, only 65 samples were detected to be positive for influenza in the present study. This trend has been reported by all countries participating in seasonal influenza surveillance at the European level. The absence of seasonal influenza epidemics had also been reported in southern hemisphere countries during their 2020 and 2021 winter seasons (25, 26). As already reported in the literature, the circulation of influenza viruses dropped drastically as soon as strict implementation of NPIs around the world was put in place in order to combat the spread of SARS-CoV-2 (27 – 33). Community hygiene measures have already been shown to reduce respiratory virus infections, as observed in Hong Kong (China) during the SARS outbreak in 2003 (34). In previous works, NPIs have been found to be effective against pandemic influenza but possibly infeasible (35). Nevertheless, these measures have had an indisputable effect against seasonal flu during SARS-CoV-2 pandemic. It was suggested that seasonal influenza have a lower reproductive number and a shorter median duration of infectivity than the original strain of SARS-CoV-2 (36, 37). Also, the immunity to seasonal influenza strains, strongly recommended during this period, reduces the speed of transmission (38). The lack of testing for influenza and other respiratory viruses during the first months of COVID-19 pandemic was due to reagent shortages, focused on SARS-CoV-2 diagnosis and completed by a reduced capacity of laboratories. All these aspects are factors that could explain the very low detection of influenza from March 2020 to September 2021.

EV/RV circulate all year round with a higher activity in the fall and the spring–summer overlap. For these viruses, airborne precautions do not seem to impact their circulation (39 – 41). Despite evidence of aerosol transmission, the transmission efficacy of RV by direct contact, fomite, and aerosols has been demonstrated and can explain the circulation of these viruses during NPI restrictions. One randomized trial in day-care nurseries suggested that biweekly disinfection of toys significantly reduced the detection of adenoviruses, EV/RV, and RSV but not common cold coronaviruses, PIV, and bocaviruses in the environment; however, surface cleaning did not reduce the incidence of respiratory illness, suggesting that transmission may have occurred via routes other than the fomite route (42).

A profound change in the circulation of RSV was seen with the significant and unusual detection of this virus in the form of two waves in 2021, later as usual during the 2020–2021 epidemics. Other studies described a delayed 2020–2021 RSV season and the inter-seasonal resurgence of respiratory viruses activity (13, 14, 29, 43). Future dynamics of endemic infections were modelized and suggested that the increase in population susceptibility due to minimal virus infections will increase circulation of RSV influenza and other respiratory viruses with large future outbreaks (32).

The COVID-19 pandemic has produced significant changes in the management of respiratory infections with a significant increase in requests for multiplex respiratory research to identify the virus responsible for the symptoms. Respiratory diagnostic needs have increased, and the last epidemic period in our study, 2021–2022, includes a number of samples analyzed to be equal to all the samples analyzed over the four epidemic periods preceding the pandemic, 15,950 samples analyzed in 2021–2022 and 15,406 samples analyzed between 2016 and 2022. We intentionally excluded the samples analyzed only for SARS-CoV-2. Nevertheless, we cannot exclude that some of multiplex prescriptions in children did not include a suspicion of SARS-CoV-2 infection.

The mechanistic effect of the COVID-19 pandemic and the interference between viruses are elements responsible for modifications in the circulation pattern of respiratory viruses. The reasons for these changes may be linked to the different modes of transmission of viruses apart from respiratory transmission and their ability to survive in the environment. Virus, host, and environmental factors influence the success of transmission by governing the infectivity of the respiratory virus, the contagiousness of the infector, the susceptibility of the exposed individual, and the environmental stress on the virus. These determinants may have different relative effects on each mode of transmission (44).

At the population level, viral interference corresponding to an ecological phenomenon in which the epidemic caused by one virus delays the onset or advances the end of the epidemic caused by another virus could influence the epidemiology of respiratory viruses. These episodes are difficult to demonstrate because the transmission dynamics of respiratory viruses might be influenced by social behaviors for different age groups (45).

There are some limitations to this study. The most important concern is the method used to calculate the percentage of positive tests. The data available did not allow us to include in the denominator of the number of tests specifically performed for the detection of each virus; thus, we used the total number of tests. As a result, the positivity rates are underestimated overall, by a proportion that we are unable to specify. However, this limitation does not apply to the influenza virus, which is systematically tested, and trends over the period studied can be analyzed without bias. Another important bias is linked to the use of different tests within laboratories and changes in diagnostic strategy over time. However, in routine laboratory practice, these strategies evolve according to the availability and development of techniques and epidemiological information. The data reported here therefore represent the “real life” activity of two French university hospital laboratories and highlight a major epidemiological change in the circulation of respiratory viruses in a pediatric population as a result of the strategies adopted to combat the COVID-19 pandemic. Other limitations relate to the retrospective nature of the study and its restriction to one region of France.

Our results focus on the importance to continue an active surveillance of respiratory viruses’ circulation and the need in new recommendations for the respiratory virus detection in pediatric patients.

## SUPPLEMENTAL MATERIAL

Figure S1 spectrum.01867-23-s0001.tif Weekly numbers of respiratory virus tests and positive tests for parainfluenzaviruses, coronaviruses and human metapneumoviruses, Normandy, France, 2016-2022.

Figure S2 spectrum.01867-23-s0002.tif Weekly numbers of respiratory virus tests and positive tests for bocaviruses and adenoviruses, Normandy, France, 2016–2022.

Table S1 spectrum.01867-23-s0003.docx Weekly data.

Table S2 spectrum.01867-23-s0004.docx Assays used and viruses detected by each test.

ASM does not own the copyrights to Supplemental Material that may be linked to, or accessed through, an article. The authors have granted ASM a non-exclusive, world-wide license to publish the Supplemental Material files. Please contact the corresponding author directly for reuse.
