# Supplementary figures and images for: Characteristics of respiratory viruses’ circulation through a six-year period (2016–2022) in a pediatric population in Normandy, France, and the impact of COVID-19 pandemic
Source: Microbiol Spectr. 2023 Oct 26;11(6):e01867-23. doi: 10.1128/spectrum.01867-23 (PMC10714951; doi:10.1128/spectrum.01867-23)

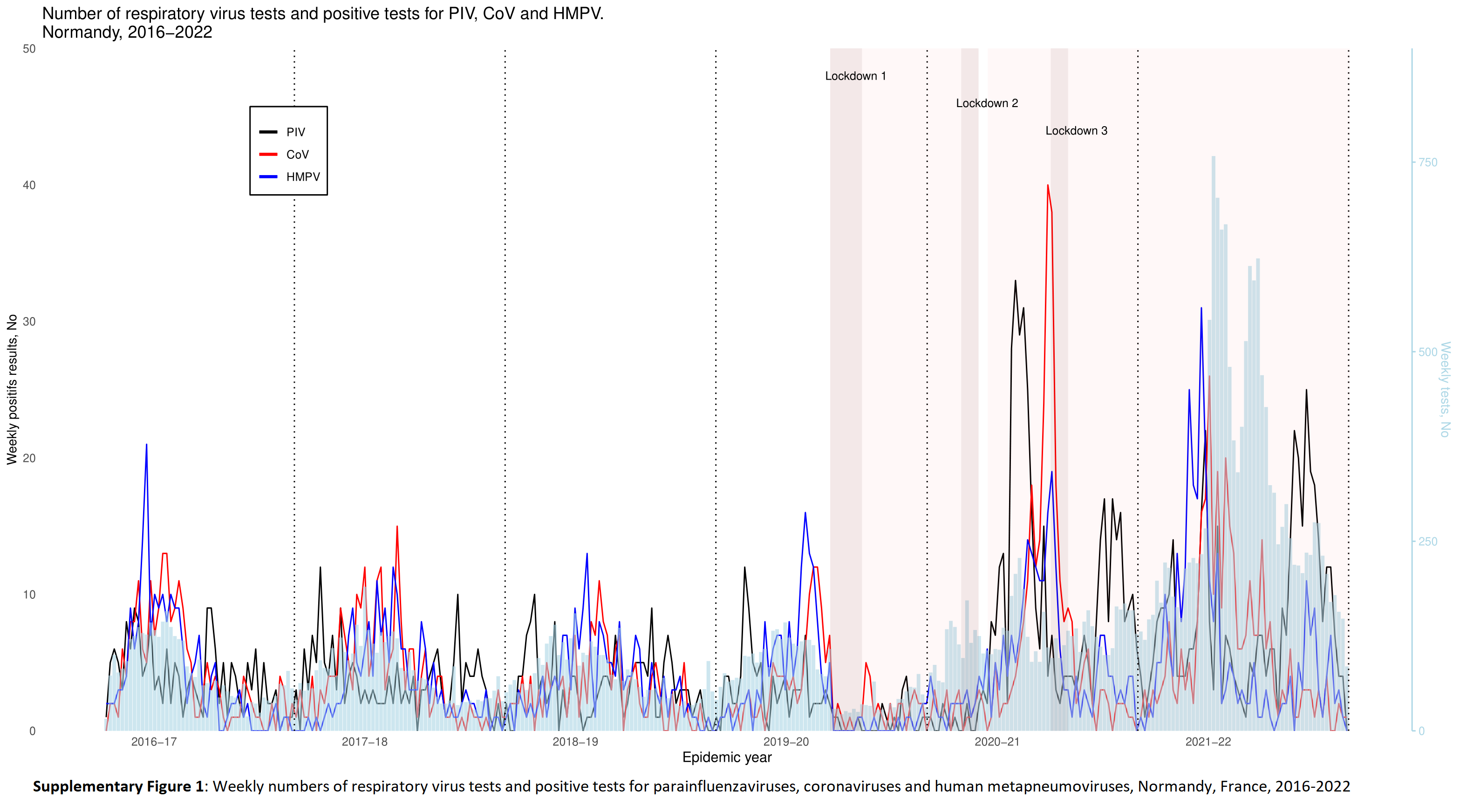

Supplement: Figure S1 — Weekly numbers of respiratory virus tests and positive tests for parainfluenzaviruses, coronaviruses and human metapneumoviruses, Normandy, France, 2016-2022. [file spectrum.01867-23-s0001.tif]

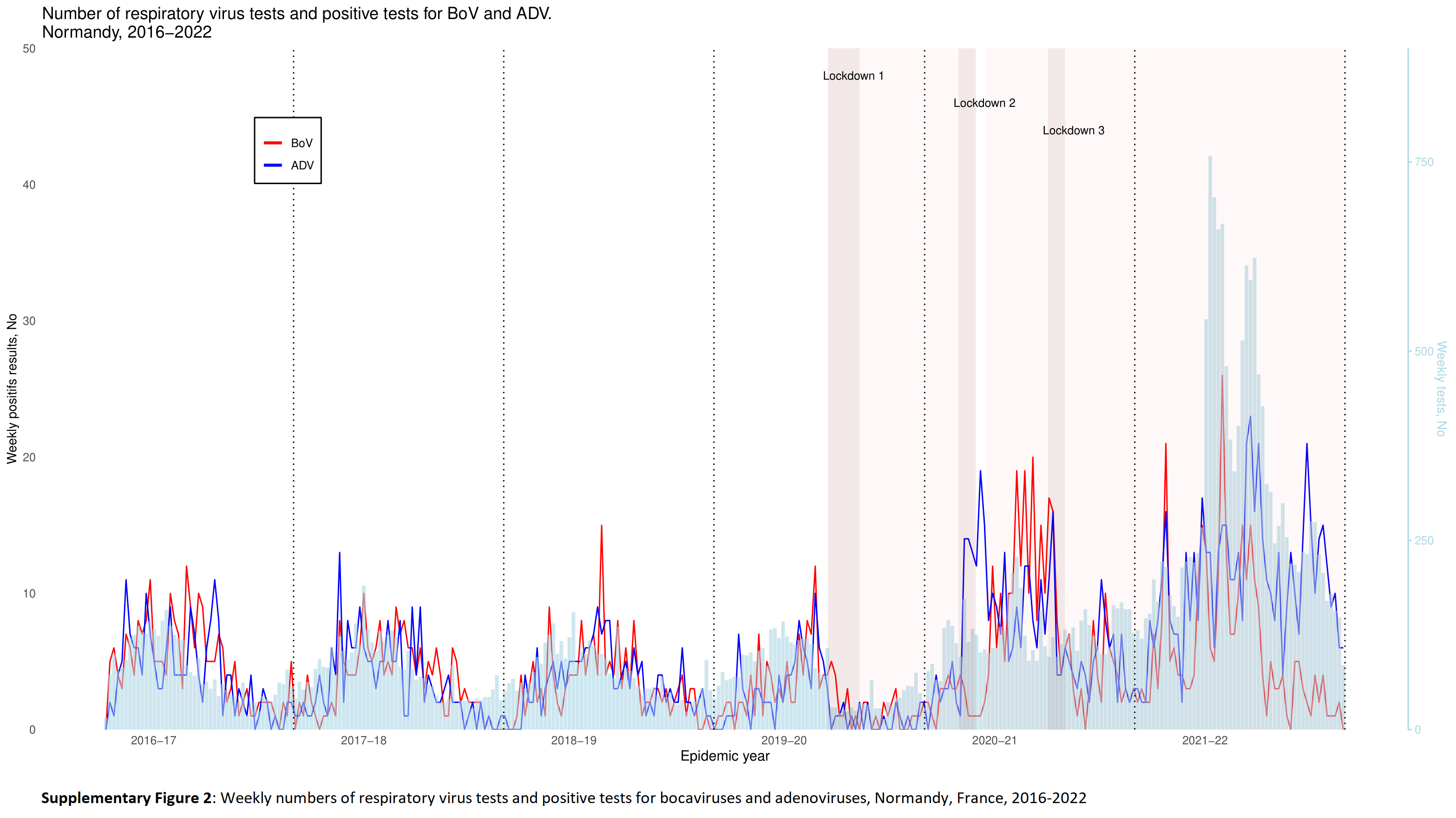

Supplement: Figure S2 — Weekly numbers of respiratory virus tests and positive tests for bocaviruses and adenoviruses, Normandy, France, 2016–2022. [file spectrum.01867-23-s0002.tif]
