# Supplementary material for: Characteristics of respiratory viruses’ circulation through a six-year period (2016–2022) in a pediatric population in Normandy, France, and the impact of COVID-19 pandemic
Source: Microbiol Spectr. 2023 Oct 26;11(6):e01867-23. doi: 10.1128/spectrum.01867-23 (PMC10714951; doi:10.1128/spectrum.01867-23)
Supplement: Table S1 — Weekly data. [file spectrum.01867-23-s0003.docx]

**Supplementary Table 1. Weekly data**

N: total number of tests,

Absolute numbers of positive tests: flu (influenza virus), rsv (respiratory syncytial virus), ev_rhv (enterovirus/rhinovirus), piv (parainfluenza virus), cov (coronavirus), hmpv (human metapneumovirus), bov (bocavirus), adv (adenovirus)

Proportion of positive tests (number of positive tests for each virus / total number of tests) : flu_p (influenza virus), rsv_p (respiratory syncytial virus), ev_rhv_p (enterovirus/rhinovirus), piv_p (parainfluenza virus), cov_p (coronavirus), hmpv_p (human metapneumovirus), bov_p (bocavirus), adv_p (adenovirus)

| **week** | **season** | **N** | **flu** | **rsv** | **ev_rhv** | **piv** | **cov** | **hmpv** | **bov** | **adv** | **flu_p** | **rsv_p** | **ev_rhv_p** | **piv_p** | **cov_p** | **hmpv_p** | **bov_p** | **adv_p** |
| --- | --- | --- | --- | --- | --- | --- | --- | --- | --- | --- | --- | --- | --- | --- | --- | --- | --- | --- |
| 2016-W41 | 2016-17 | 16 | 0 | 1 | 7 | 1 | 0 | 2 | 0 | 0 | 0.0 | 6.2 | 43.8 | 6.2 | 0.0 | 12.5 | 0.0 | 0.0 |
| 2016-W42 | 2016-17 | 73 | 2 | 15 | 23 | 5 | 2 | 2 | 5 | 2 | 2.7 | 20.5 | 31.5 | 6.8 | 2.7 | 2.7 | 6.8 | 2.7 |
| 2016-W43 | 2016-17 | 100 | 3 | 16 | 48 | 6 | 2 | 2 | 6 | 1 | 3.0 | 16.0 | 48.0 | 6.0 | 2.0 | 2.0 | 6.0 | 1.0 |
| 2016-W44 | 2016-17 | 77 | 0 | 18 | 33 | 5 | 1 | 3 | 4 | 4 | 0.0 | 23.4 | 42.9 | 6.5 | 1.3 | 3.9 | 5.2 | 5.2 |
| 2016-W45 | 2016-17 | 82 | 1 | 15 | 40 | 3 | 6 | 3 | 3 | 5 | 1.2 | 18.3 | 48.8 | 3.7 | 7.3 | 3.7 | 3.7 | 6.1 |
| 2016-W46 | 2016-17 | 92 | 3 | 22 | 45 | 8 | 4 | 4 | 7 | 11 | 3.3 | 23.9 | 48.9 | 8.7 | 4.3 | 4.3 | 7.6 | 12.0 |
| 2016-W47 | 2016-17 | 121 | 2 | 32 | 52 | 6 | 8 | 9 | 6 | 7 | 1.7 | 26.4 | 43.0 | 5.0 | 6.6 | 7.4 | 5.0 | 5.8 |
| 2016-W48 | 2016-17 | 125 | 3 | 38 | 47 | 9 | 8 | 6 | 4 | 6 | 2.4 | 30.4 | 37.6 | 7.2 | 6.4 | 4.8 | 3.2 | 4.8 |
| 2016-W49 | 2016-17 | 137 | 3 | 55 | 43 | 8 | 11 | 8 | 8 | 6 | 2.2 | 40.1 | 31.4 | 5.8 | 8.0 | 5.8 | 5.8 | 4.4 |
| 2016-W50 | 2016-17 | 129 | 2 | 20 | 43 | 4 | 6 | 14 | 7 | 4 | 1.6 | 15.5 | 33.3 | 3.1 | 4.7 | 10.9 | 5.4 | 3.1 |
| 2016-W51 | 2016-17 | 127 | 3 | 30 | 38 | 5 | 5 | 21 | 8 | 10 | 2.4 | 23.6 | 29.9 | 3.9 | 3.9 | 16.5 | 6.3 | 7.9 |
| 2016-W52 | 2016-17 | 143 | 5 | 39 | 43 | 8 | 11 | 7 | 11 | 7 | 3.5 | 27.3 | 30.1 | 5.6 | 7.7 | 4.9 | 7.7 | 4.9 |
| 2017-W01 | 2016-17 | 133 | 7 | 30 | 40 | 3 | 7 | 10 | 5 | 5 | 5.3 | 22.6 | 30.1 | 2.3 | 5.3 | 7.5 | 3.8 | 3.8 |
| 2017-W02 | 2016-17 | 124 | 11 | 18 | 31 | 4 | 9 | 9 | 5 | 3 | 8.9 | 14.5 | 25.0 | 3.2 | 7.3 | 7.3 | 4.0 | 2.4 |
| 2017-W03 | 2016-17 | 144 | 15 | 21 | 32 | 2 | 13 | 10 | 5 | 3 | 10.4 | 14.6 | 22.2 | 1.4 | 9.0 | 6.9 | 3.5 | 2.1 |
| 2017-W04 | 2016-17 | 158 | 8 | 38 | 48 | 6 | 13 | 8 | 4 | 6 | 5.1 | 24.1 | 30.4 | 3.8 | 8.2 | 5.1 | 2.5 | 3.8 |
| 2017-W05 | 2016-17 | 137 | 15 | 17 | 49 | 2 | 8 | 10 | 10 | 9 | 10.9 | 12.4 | 35.8 | 1.5 | 5.8 | 7.3 | 7.3 | 6.6 |
| 2017-W06 | 2016-17 | 125 | 12 | 24 | 39 | 5 | 9 | 9 | 8 | 4 | 9.6 | 19.2 | 31.2 | 4.0 | 7.2 | 7.2 | 6.4 | 3.2 |
| 2017-W07 | 2016-17 | 121 | 10 | 25 | 47 | 4 | 11 | 9 | 7 | 4 | 8.3 | 20.7 | 38.8 | 3.3 | 9.1 | 7.4 | 5.8 | 3.3 |
| 2017-W08 | 2016-17 | 119 | 6 | 16 | 38 | 1 | 9 | 6 | 3 | 4 | 5.0 | 13.4 | 31.9 | 0.8 | 7.6 | 5.0 | 2.5 | 3.4 |
| 2017-W09 | 2016-17 | 85 | 2 | 7 | 24 | 3 | 6 | 2 | 12 | 4 | 2.4 | 8.2 | 28.2 | 3.5 | 7.1 | 2.4 | 14.1 | 4.7 |
| 2017-W10 | 2016-17 | 76 | 1 | 14 | 30 | 4 | 5 | 4 | 9 | 9 | 1.3 | 18.4 | 39.5 | 5.3 | 6.6 | 5.3 | 11.8 | 11.8 |
| 2017-W11 | 2016-17 | 71 | 3 | 5 | 39 | 3 | 1 | 5 | 6 | 7 | 4.2 | 7.0 | 54.9 | 4.2 | 1.4 | 7.0 | 8.5 | 9.9 |
| 2017-W12 | 2016-17 | 94 | 3 | 11 | 39 | 2 | 1 | 7 | 10 | 4 | 3.2 | 11.7 | 41.5 | 2.1 | 1.1 | 7.4 | 10.6 | 4.3 |
| 2017-W13 | 2016-17 | 60 | 1 | 4 | 42 | 1 | 3 | 2 | 9 | 2 | 1.7 | 6.7 | 70.0 | 1.7 | 5.0 | 3.3 | 15.0 | 3.3 |
| 2017-W14 | 2016-17 | 63 | 0 | 7 | 35 | 9 | 5 | 1 | 5 | 6 | 0.0 | 11.1 | 55.6 | 14.3 | 7.9 | 1.6 | 7.9 | 9.5 |
| 2017-W15 | 2016-17 | 54 | 3 | 7 | 32 | 9 | 3 | 4 | 5 | 8 | 5.6 | 13.0 | 59.3 | 16.7 | 5.6 | 7.4 | 9.3 | 14.8 |
| 2017-W16 | 2016-17 | 66 | 0 | 8 | 31 | 6 | 4 | 5 | 5 | 11 | 0.0 | 12.1 | 47.0 | 9.1 | 6.1 | 7.6 | 7.6 | 16.7 |
| 2017-W17 | 2016-17 | 44 | 1 | 5 | 41 | 2 | 3 | 0 | 7 | 8 | 2.3 | 11.4 | 93.2 | 4.5 | 6.8 | 0.0 | 15.9 | 18.2 |
| 2017-W18 | 2016-17 | 60 | 0 | 4 | 22 | 5 | 1 | 0 | 6 | 1 | 0.0 | 6.7 | 36.7 | 8.3 | 1.7 | 0.0 | 10.0 | 1.7 |
| 2017-W19 | 2016-17 | 52 | 1 | 5 | 29 | 1 | 0 | 1 | 2 | 4 | 1.9 | 9.6 | 55.8 | 1.9 | 0.0 | 1.9 | 3.8 | 7.7 |
| 2017-W20 | 2016-17 | 46 | 0 | 1 | 25 | 5 | 1 | 2 | 3 | 4 | 0.0 | 2.2 | 54.3 | 10.9 | 2.2 | 4.3 | 6.5 | 8.7 |
| 2017-W21 | 2016-17 | 44 | 1 | 1 | 20 | 4 | 1 | 2 | 5 | 1 | 2.3 | 2.3 | 45.5 | 9.1 | 2.3 | 4.5 | 11.4 | 2.3 |
| 2017-W22 | 2016-17 | 36 | 0 | 8 | 18 | 2 | 1 | 3 | 1 | 3 | 0.0 | 22.2 | 50.0 | 5.6 | 2.8 | 8.3 | 2.8 | 8.3 |
| 2017-W23 | 2016-17 | 62 | 0 | 4 | 19 | 1 | 4 | 1 | 2 | 2 | 0.0 | 6.5 | 30.6 | 1.6 | 6.5 | 1.6 | 3.2 | 3.2 |
| 2017-W24 | 2016-17 | 19 | 0 | 2 | 11 | 5 | 3 | 2 | 3 | 1 | 0.0 | 10.5 | 57.9 | 26.3 | 15.8 | 10.5 | 15.8 | 5.3 |
| 2017-W25 | 2016-17 | 38 | 0 | 3 | 20 | 3 | 2 | 0 | 1 | 4 | 0.0 | 7.9 | 52.6 | 7.9 | 5.3 | 0.0 | 2.6 | 10.5 |
| 2017-W26 | 2016-17 | 36 | 1 | 2 | 27 | 6 | 1 | 0 | 1 | 0 | 2.8 | 5.6 | 75.0 | 16.7 | 2.8 | 0.0 | 2.8 | 0.0 |
| 2017-W27 | 2016-17 | 24 | 0 | 1 | 18 | 1 | 1 | 0 | 2 | 1 | 0.0 | 4.2 | 75.0 | 4.2 | 4.2 | 0.0 | 8.3 | 4.2 |
| 2017-W28 | 2016-17 | 44 | 0 | 8 | 18 | 5 | 0 | 0 | 2 | 3 | 0.0 | 18.2 | 40.9 | 11.4 | 0.0 | 0.0 | 4.5 | 6.8 |
| 2017-W29 | 2016-17 | 40 | 1 | 6 | 10 | 2 | 0 | 0 | 2 | 2 | 2.5 | 15.0 | 25.0 | 5.0 | 0.0 | 0.0 | 5.0 | 5.0 |
| 2017-W30 | 2016-17 | 37 | 1 | 5 | 8 | 2 | 2 | 1 | 2 | 0 | 2.7 | 13.5 | 21.6 | 5.4 | 5.4 | 2.7 | 5.4 | 0.0 |
| 2017-W31 | 2016-17 | 46 | 1 | 4 | 15 | 3 | 0 | 2 | 1 | 1 | 2.2 | 8.7 | 32.6 | 6.5 | 0.0 | 4.3 | 2.2 | 2.2 |
| 2017-W32 | 2016-17 | 62 | 1 | 3 | 5 | 0 | 4 | 0 | 0 | 0 | 1.6 | 4.8 | 8.1 | 0.0 | 6.5 | 0.0 | 0.0 | 0.0 |
| 2017-W33 | 2016-17 | 60 | 0 | 6 | 13 | 1 | 3 | 1 | 2 | 0 | 0.0 | 10.0 | 21.7 | 1.7 | 5.0 | 1.7 | 3.3 | 0.0 |
| 2017-W34 | 2016-17 | 79 | 0 | 3 | 7 | 1 | 0 | 1 | 1 | 2 | 0.0 | 3.8 | 8.9 | 1.3 | 0.0 | 1.3 | 1.3 | 2.5 |
| 2017-W35 | 2016-17 | 56 | 0 | 3 | 4 | 1 | 2 | 0 | 5 | 2 | 0.0 | 5.4 | 7.1 | 1.8 | 3.6 | 0.0 | 8.9 | 3.6 |
| 2017-W36 | 2017-18 | 41 | 0 | 3 | 6 | 3 | 1 | 0 | 0 | 1 | 0.0 | 7.3 | 14.6 | 7.3 | 2.4 | 0.0 | 0.0 | 2.4 |
| 2017-W37 | 2017-18 | 63 | 2 | 5 | 19 | 0 | 3 | 0 | 2 | 1 | 3.2 | 7.9 | 30.2 | 0.0 | 4.8 | 0.0 | 3.2 | 1.6 |
| 2017-W38 | 2017-18 | 53 | 0 | 3 | 46 | 6 | 1 | 0 | 1 | 2 | 0.0 | 5.7 | 86.8 | 11.3 | 1.9 | 0.0 | 1.9 | 3.8 |
| 2017-W39 | 2017-18 | 63 | 0 | 3 | 32 | 3 | 1 | 1 | 4 | 1 | 0.0 | 4.8 | 50.8 | 4.8 | 1.6 | 1.6 | 6.3 | 1.6 |
| 2017-W40 | 2017-18 | 56 | 1 | 6 | 20 | 7 | 4 | 0 | 2 | 1 | 1.8 | 10.7 | 35.7 | 12.5 | 7.1 | 0.0 | 3.6 | 1.8 |
| 2017-W41 | 2017-18 | 80 | 1 | 8 | 23 | 5 | 0 | 1 | 1 | 2 | 1.2 | 10.0 | 28.7 | 6.2 | 0.0 | 1.2 | 1.2 | 2.5 |
| 2017-W42 | 2017-18 | 93 | 0 | 9 | 24 | 12 | 3 | 0 | 0 | 4 | 0.0 | 9.7 | 25.8 | 12.9 | 3.2 | 0.0 | 0.0 | 4.3 |
| 2017-W43 | 2017-18 | 83 | 1 | 11 | 25 | 5 | 2 | 1 | 1 | 2 | 1.2 | 13.3 | 30.1 | 6.0 | 2.4 | 1.2 | 1.2 | 2.4 |
| 2017-W44 | 2017-18 | 82 | 1 | 8 | 21 | 4 | 4 | 2 | 1 | 1 | 1.2 | 9.8 | 25.6 | 4.9 | 4.9 | 2.4 | 1.2 | 1.2 |
| 2017-W45 | 2017-18 | 109 | 1 | 12 | 22 | 7 | 4 | 1 | 2 | 6 | 0.9 | 11.0 | 20.2 | 6.4 | 3.7 | 0.9 | 1.8 | 5.5 |
| 2017-W46 | 2017-18 | 73 | 2 | 20 | 19 | 3 | 4 | 2 | 1 | 4 | 2.7 | 27.4 | 26.0 | 4.1 | 5.5 | 2.7 | 1.4 | 5.5 |
| 2017-W47 | 2017-18 | 123 | 3 | 32 | 55 | 9 | 9 | 2 | 8 | 13 | 2.4 | 26.0 | 44.7 | 7.3 | 7.3 | 1.6 | 6.5 | 10.6 |
| 2017-W48 | 2017-18 | 104 | 3 | 22 | 29 | 2 | 7 | 3 | 5 | 2 | 2.9 | 21.2 | 27.9 | 1.9 | 6.7 | 2.9 | 4.8 | 1.9 |
| 2017-W49 | 2017-18 | 93 | 9 | 21 | 43 | 4 | 5 | 7 | 4 | 8 | 9.7 | 22.6 | 46.2 | 4.3 | 5.4 | 7.5 | 4.3 | 8.6 |
| 2017-W50 | 2017-18 | 102 | 9 | 17 | 28 | 5 | 3 | 9 | 4 | 6 | 8.8 | 16.7 | 27.5 | 4.9 | 2.9 | 8.8 | 3.9 | 5.9 |
| 2017-W51 | 2017-18 | 140 | 16 | 27 | 37 | 4 | 10 | 5 | 4 | 6 | 11.4 | 19.3 | 26.4 | 2.9 | 7.1 | 3.6 | 2.9 | 4.3 |
| 2017-W52 | 2017-18 | 112 | 23 | 22 | 26 | 2 | 9 | 4 | 6 | 9 | 20.5 | 19.6 | 23.2 | 1.8 | 8.0 | 3.6 | 5.4 | 8.0 |
| 2018-W01 | 2017-18 | 190 | 40 | 41 | 42 | 3 | 12 | 6 | 10 | 6 | 21.1 | 21.6 | 22.1 | 1.6 | 6.3 | 3.2 | 5.3 | 3.2 |
| 2018-W02 | 2017-18 | 133 | 10 | 28 | 32 | 2 | 4 | 8 | 6 | 5 | 7.5 | 21.1 | 24.1 | 1.5 | 3.0 | 6.0 | 4.5 | 3.8 |
| 2018-W03 | 2017-18 | 115 | 12 | 29 | 22 | 3 | 6 | 4 | 5 | 5 | 10.4 | 25.2 | 19.1 | 2.6 | 5.2 | 3.5 | 4.3 | 4.3 |
| 2018-W04 | 2017-18 | 103 | 7 | 13 | 30 | 2 | 11 | 11 | 6 | 3 | 6.8 | 12.6 | 29.1 | 1.9 | 10.7 | 10.7 | 5.8 | 2.9 |
| 2018-W05 | 2017-18 | 130 | 13 | 16 | 44 | 2 | 12 | 7 | 8 | 5 | 10.0 | 12.3 | 33.8 | 1.5 | 9.2 | 5.4 | 6.2 | 3.8 |
| 2018-W06 | 2017-18 | 117 | 10 | 25 | 48 | 2 | 3 | 9 | 4 | 5 | 8.5 | 21.4 | 41.0 | 1.7 | 2.6 | 7.7 | 3.4 | 4.3 |
| 2018-W07 | 2017-18 | 125 | 2 | 6 | 47 | 4 | 5 | 5 | 5 | 8 | 1.6 | 4.8 | 37.6 | 3.2 | 4.0 | 4.0 | 4.0 | 6.4 |
| 2018-W08 | 2017-18 | 131 | 9 | 18 | 43 | 2 | 6 | 12 | 4 | 5 | 6.9 | 13.7 | 32.8 | 1.5 | 4.6 | 9.2 | 3.1 | 3.8 |
| 2018-W09 | 2017-18 | 122 | 8 | 13 | 49 | 5 | 15 | 10 | 9 | 5 | 6.6 | 10.7 | 40.2 | 4.1 | 12.3 | 8.2 | 7.4 | 4.1 |
| 2018-W10 | 2017-18 | 83 | 13 | 7 | 32 | 2 | 8 | 6 | 7 | 8 | 15.7 | 8.4 | 38.6 | 2.4 | 9.6 | 7.2 | 8.4 | 9.6 |
| 2018-W11 | 2017-18 | 79 | 5 | 12 | 28 | 3 | 3 | 6 | 8 | 1 | 6.3 | 15.2 | 35.4 | 3.8 | 3.8 | 7.6 | 10.1 | 1.3 |
| 2018-W12 | 2017-18 | 104 | 8 | 7 | 28 | 2 | 6 | 3 | 6 | 1 | 7.7 | 6.7 | 26.9 | 1.9 | 5.8 | 2.9 | 5.8 | 1.0 |
| 2018-W13 | 2017-18 | 80 | 9 | 12 | 33 | 4 | 6 | 3 | 6 | 9 | 11.2 | 15.0 | 41.2 | 5.0 | 7.5 | 3.8 | 7.5 | 11.2 |
| 2018-W14 | 2017-18 | 66 | 8 | 5 | 32 | 0 | 1 | 3 | 4 | 4 | 12.1 | 7.6 | 48.5 | 0.0 | 1.5 | 4.5 | 6.1 | 6.1 |
| 2018-W15 | 2017-18 | 70 | 0 | 15 | 31 | 3 | 4 | 8 | 6 | 9 | 0.0 | 21.4 | 44.3 | 4.3 | 5.7 | 11.4 | 8.6 | 12.9 |
| 2018-W16 | 2017-18 | 68 | 3 | 8 | 43 | 3 | 6 | 6 | 3 | 2 | 4.4 | 11.8 | 63.2 | 4.4 | 8.8 | 8.8 | 4.4 | 2.9 |
| 2018-W17 | 2017-18 | 60 | 2 | 1 | 25 | 4 | 1 | 2 | 5 | 4 | 3.3 | 1.7 | 41.7 | 6.7 | 1.7 | 3.3 | 8.3 | 6.7 |
| 2018-W18 | 2017-18 | 57 | 3 | 6 | 23 | 5 | 2 | 5 | 4 | 3 | 5.3 | 10.5 | 40.4 | 8.8 | 3.5 | 8.8 | 7.0 | 5.3 |
| 2018-W19 | 2017-18 | 45 | 0 | 2 | 12 | 6 | 4 | 2 | 6 | 2 | 0.0 | 4.4 | 26.7 | 13.3 | 8.9 | 4.4 | 13.3 | 4.4 |
| 2018-W20 | 2017-18 | 36 | 1 | 4 | 19 | 3 | 4 | 3 | 4 | 2 | 2.8 | 11.1 | 52.8 | 8.3 | 11.1 | 8.3 | 11.1 | 5.6 |
| 2018-W21 | 2017-18 | 36 | 0 | 2 | 12 | 1 | 1 | 1 | 1 | 3 | 0.0 | 5.6 | 33.3 | 2.8 | 2.8 | 2.8 | 2.8 | 8.3 |
| 2018-W22 | 2017-18 | 40 | 0 | 3 | 14 | 2 | 1 | 2 | 1 | 4 | 0.0 | 7.5 | 35.0 | 5.0 | 2.5 | 5.0 | 2.5 | 10.0 |
| 2018-W23 | 2017-18 | 85 | 1 | 5 | 25 | 5 | 2 | 1 | 6 | 2 | 1.2 | 5.9 | 29.4 | 5.9 | 2.4 | 1.2 | 7.1 | 2.4 |
| 2018-W24 | 2017-18 | 34 | 1 | 1 | 26 | 10 | 0 | 1 | 5 | 2 | 2.9 | 2.9 | 76.5 | 29.4 | 0.0 | 2.9 | 14.7 | 5.9 |
| 2018-W25 | 2017-18 | 38 | 1 | 3 | 19 | 2 | 1 | 2 | 2 | 2 | 2.6 | 7.9 | 50.0 | 5.3 | 2.6 | 5.3 | 5.3 | 5.3 |
| 2018-W26 | 2017-18 | 38 | 3 | 3 | 21 | 5 | 0 | 3 | 3 | 0 | 7.9 | 7.9 | 55.3 | 13.2 | 0.0 | 7.9 | 7.9 | 0.0 |
| 2018-W27 | 2017-18 | 40 | 0 | 1 | 16 | 4 | 2 | 2 | 2 | 1 | 0.0 | 2.5 | 40.0 | 10.0 | 5.0 | 5.0 | 5.0 | 2.5 |
| 2018-W28 | 2017-18 | 28 | 1 | 5 | 19 | 4 | 1 | 2 | 2 | 2 | 3.6 | 17.9 | 67.9 | 14.3 | 3.6 | 7.1 | 7.1 | 7.1 |
| 2018-W29 | 2017-18 | 41 | 0 | 2 | 17 | 6 | 1 | 1 | 2 | 0 | 0.0 | 4.9 | 41.5 | 14.6 | 2.4 | 2.4 | 4.9 | 0.0 |
| 2018-W30 | 2017-18 | 39 | 1 | 2 | 5 | 4 | 0 | 1 | 2 | 2 | 2.6 | 5.1 | 12.8 | 10.3 | 0.0 | 2.6 | 5.1 | 5.1 |
| 2018-W31 | 2017-18 | 43 | 1 | 3 | 6 | 3 | 1 | 3 | 0 | 0 | 2.3 | 7.0 | 14.0 | 7.0 | 2.3 | 7.0 | 0.0 | 0.0 |
| 2018-W32 | 2017-18 | 43 | 0 | 3 | 6 | 1 | 0 | 0 | 1 | 1 | 0.0 | 7.0 | 14.0 | 2.3 | 0.0 | 0.0 | 2.3 | 2.3 |
| 2018-W33 | 2017-18 | 53 | 0 | 1 | 7 | 0 | 1 | 1 | 0 | 0 | 0.0 | 1.9 | 13.2 | 0.0 | 1.9 | 1.9 | 0.0 | 0.0 |
| 2018-W34 | 2017-18 | 72 | 1 | 1 | 7 | 2 | 3 | 1 | 0 | 0 | 1.4 | 1.4 | 9.7 | 2.8 | 4.2 | 1.4 | 0.0 | 0.0 |
| 2018-W35 | 2017-18 | 39 | 0 | 0 | 4 | 0 | 1 | 0 | 0 | 1 | 0.0 | 0.0 | 10.3 | 0.0 | 2.6 | 0.0 | 0.0 | 2.6 |
| 2018-W36 | 2018-19 | 51 | 1 | 2 | 8 | 2 | 1 | 1 | 0 | 1 | 2.0 | 3.9 | 15.7 | 3.9 | 2.0 | 2.0 | 0.0 | 2.0 |
| 2018-W37 | 2018-19 | 61 | 2 | 3 | 20 | 0 | 2 | 2 | 0 | 0 | 3.3 | 4.9 | 32.8 | 0.0 | 3.3 | 3.3 | 0.0 | 0.0 |
| 2018-W38 | 2018-19 | 67 | 2 | 5 | 36 | 0 | 2 | 0 | 0 | 0 | 3.0 | 7.5 | 53.7 | 0.0 | 3.0 | 0.0 | 0.0 | 0.0 |
| 2018-W39 | 2018-19 | 51 | 1 | 6 | 34 | 4 | 2 | 3 | 1 | 0 | 2.0 | 11.8 | 66.7 | 7.8 | 3.9 | 5.9 | 2.0 | 0.0 |
| 2018-W40 | 2018-19 | 62 | 0 | 5 | 39 | 4 | 4 | 2 | 4 | 0 | 0.0 | 8.1 | 62.9 | 6.5 | 6.5 | 3.2 | 6.5 | 0.0 |
| 2018-W41 | 2018-19 | 54 | 1 | 3 | 26 | 7 | 1 | 1 | 1 | 4 | 1.9 | 5.6 | 48.1 | 13.0 | 1.9 | 1.9 | 1.9 | 7.4 |
| 2018-W42 | 2018-19 | 62 | 1 | 3 | 18 | 8 | 4 | 2 | 3 | 2 | 1.6 | 4.8 | 29.0 | 12.9 | 6.5 | 3.2 | 4.8 | 3.2 |
| 2018-W43 | 2018-19 | 76 | 0 | 9 | 29 | 10 | 0 | 1 | 5 | 2 | 0.0 | 11.8 | 38.2 | 13.2 | 0.0 | 1.3 | 6.6 | 2.6 |
| 2018-W44 | 2018-19 | 96 | 1 | 9 | 21 | 3 | 3 | 2 | 2 | 6 | 1.0 | 9.4 | 21.9 | 3.1 | 3.1 | 2.1 | 2.1 | 6.2 |
| 2018-W45 | 2018-19 | 87 | 1 | 14 | 20 | 4 | 1 | 4 | 3 | 1 | 1.1 | 16.1 | 23.0 | 4.6 | 1.1 | 4.6 | 3.4 | 1.1 |
| 2018-W46 | 2018-19 | 115 | 1 | 12 | 21 | 2 | 5 | 1 | 1 | 3 | 0.9 | 10.4 | 18.3 | 1.7 | 4.3 | 0.9 | 0.9 | 2.6 |
| 2018-W47 | 2018-19 | 125 | 2 | 17 | 22 | 3 | 3 | 3 | 9 | 4 | 1.6 | 13.6 | 17.6 | 2.4 | 2.4 | 2.4 | 7.2 | 3.2 |
| 2018-W48 | 2018-19 | 140 | 1 | 27 | 28 | 8 | 4 | 4 | 4 | 5 | 0.7 | 19.3 | 20.0 | 5.7 | 2.9 | 2.9 | 2.9 | 3.6 |
| 2018-W49 | 2018-19 | 100 | 1 | 51 | 19 | 0 | 3 | 3 | 2 | 3 | 1.0 | 51.0 | 19.0 | 0.0 | 3.0 | 3.0 | 2.0 | 3.0 |
| 2018-W50 | 2018-19 | 117 | 1 | 61 | 33 | 3 | 4 | 7 | 1 | 5 | 0.9 | 52.1 | 28.2 | 2.6 | 3.4 | 6.0 | 0.9 | 4.3 |
| 2018-W51 | 2018-19 | 94 | 5 | 75 | 21 | 1 | 1 | 7 | 3 | 3 | 5.3 | 79.8 | 22.3 | 1.1 | 1.1 | 7.4 | 3.2 | 3.2 |
| 2018-W52 | 2018-19 | 122 | 5 | 55 | 22 | 4 | 4 | 3 | 4 | 5 | 4.1 | 45.1 | 18.0 | 3.3 | 3.3 | 2.5 | 3.3 | 4.1 |
| 2019-W01 | 2018-19 | 155 | 6 | 64 | 20 | 4 | 7 | 9 | 4 | 5 | 3.9 | 41.3 | 12.9 | 2.6 | 4.5 | 5.8 | 2.6 | 3.2 |
| 2019-W02 | 2018-19 | 86 | 3 | 38 | 26 | 2 | 1 | 6 | 4 | 5 | 3.5 | 44.2 | 30.2 | 2.3 | 1.2 | 7.0 | 4.7 | 5.8 |
| 2019-W03 | 2018-19 | 103 | 9 | 23 | 30 | 0 | 5 | 9 | 8 | 5 | 8.7 | 22.3 | 29.1 | 0.0 | 4.9 | 8.7 | 7.8 | 4.9 |
| 2019-W04 | 2018-19 | 100 | 14 | 30 | 28 | 1 | 2 | 13 | 4 | 6 | 14.0 | 30.0 | 28.0 | 1.0 | 2.0 | 13.0 | 4.0 | 6.0 |
| 2019-W05 | 2018-19 | 118 | 25 | 21 | 41 | 1 | 8 | 5 | 6 | 6 | 21.2 | 17.8 | 34.7 | 0.8 | 6.8 | 4.2 | 5.1 | 5.1 |
| 2019-W06 | 2018-19 | 111 | 45 | 12 | 31 | 2 | 7 | 1 | 7 | 7 | 40.5 | 10.8 | 27.9 | 1.8 | 6.3 | 0.9 | 6.3 | 6.3 |
| 2019-W07 | 2018-19 | 100 | 27 | 17 | 35 | 3 | 11 | 8 | 4 | 9 | 27.0 | 17.0 | 35.0 | 3.0 | 11.0 | 8.0 | 4.0 | 9.0 |
| 2019-W08 | 2018-19 | 100 | 21 | 14 | 27 | 4 | 8 | 7 | 15 | 7 | 21.0 | 14.0 | 27.0 | 4.0 | 8.0 | 7.0 | 15.0 | 7.0 |
| 2019-W09 | 2018-19 | 94 | 7 | 10 | 27 | 4 | 7 | 5 | 4 | 8 | 7.4 | 10.6 | 28.7 | 4.3 | 7.4 | 5.3 | 4.3 | 8.5 |
| 2019-W10 | 2018-19 | 74 | 9 | 5 | 25 | 3 | 3 | 5 | 5 | 8 | 12.2 | 6.8 | 33.8 | 4.1 | 4.1 | 6.8 | 6.8 | 10.8 |
| 2019-W11 | 2018-19 | 68 | 4 | 6 | 23 | 7 | 6 | 4 | 4 | 3 | 5.9 | 8.8 | 33.8 | 10.3 | 8.8 | 5.9 | 5.9 | 4.4 |
| 2019-W12 | 2018-19 | 136 | 5 | 15 | 34 | 4 | 7 | 8 | 8 | 3 | 3.7 | 11.0 | 25.0 | 2.9 | 5.1 | 5.9 | 5.9 | 2.2 |
| 2019-W13 | 2018-19 | 66 | 2 | 3 | 43 | 1 | 0 | 3 | 3 | 4 | 3.0 | 4.5 | 65.2 | 1.5 | 0.0 | 4.5 | 4.5 | 6.1 |
| 2019-W14 | 2018-19 | 81 | 0 | 9 | 34 | 2 | 3 | 4 | 6 | 5 | 0.0 | 11.1 | 42.0 | 2.5 | 3.7 | 4.9 | 7.4 | 6.2 |
| 2019-W15 | 2018-19 | 78 | 1 | 7 | 28 | 4 | 4 | 4 | 3 | 3 | 1.3 | 9.0 | 35.9 | 5.1 | 5.1 | 5.1 | 3.8 | 3.8 |
| 2019-W16 | 2018-19 | 68 | 0 | 2 | 25 | 5 | 5 | 7 | 8 | 6 | 0.0 | 2.9 | 36.8 | 7.4 | 7.4 | 10.3 | 11.8 | 8.8 |
| 2019-W17 | 2018-19 | 58 | 1 | 3 | 18 | 5 | 2 | 6 | 4 | 4 | 1.7 | 5.2 | 31.0 | 8.6 | 3.4 | 10.3 | 6.9 | 6.9 |
| 2019-W18 | 2018-19 | 45 | 0 | 2 | 16 | 5 | 1 | 1 | 1 | 5 | 0.0 | 4.4 | 35.6 | 11.1 | 2.2 | 2.2 | 2.2 | 11.1 |
| 2019-W19 | 2018-19 | 54 | 2 | 2 | 23 | 4 | 1 | 2 | 2 | 1 | 3.7 | 3.7 | 42.6 | 7.4 | 1.9 | 3.7 | 3.7 | 1.9 |
| 2019-W20 | 2018-19 | 54 | 0 | 1 | 28 | 9 | 4 | 5 | 2 | 2 | 0.0 | 1.9 | 51.9 | 16.7 | 7.4 | 9.3 | 3.7 | 3.7 |
| 2019-W21 | 2018-19 | 56 | 0 | 3 | 28 | 1 | 5 | 3 | 3 | 1 | 0.0 | 5.4 | 50.0 | 1.8 | 8.9 | 5.4 | 5.4 | 1.8 |
| 2019-W22 | 2018-19 | 45 | 1 | 6 | 32 | 1 | 3 | 2 | 3 | 4 | 2.2 | 13.3 | 71.1 | 2.2 | 6.7 | 4.4 | 6.7 | 8.9 |
| 2019-W23 | 2018-19 | 70 | 1 | 2 | 17 | 5 | 0 | 4 | 2 | 4 | 1.4 | 2.9 | 24.3 | 7.1 | 0.0 | 5.7 | 2.9 | 5.7 |
| 2019-W24 | 2018-19 | 43 | 0 | 3 | 23 | 7 | 0 | 1 | 3 | 2 | 0.0 | 7.0 | 53.5 | 16.3 | 0.0 | 2.3 | 7.0 | 4.7 |
| 2019-W25 | 2018-19 | 49 | 0 | 3 | 16 | 5 | 3 | 3 | 1 | 3 | 0.0 | 6.1 | 32.7 | 10.2 | 6.1 | 6.1 | 2.0 | 6.1 |
| 2019-W26 | 2018-19 | 36 | 1 | 3 | 27 | 2 | 0 | 3 | 2 | 2 | 2.8 | 8.3 | 75.0 | 5.6 | 0.0 | 8.3 | 5.6 | 5.6 |
| 2019-W27 | 2018-19 | 33 | 1 | 4 | 20 | 3 | 2 | 4 | 3 | 2 | 3.0 | 12.1 | 60.6 | 9.1 | 6.1 | 12.1 | 9.1 | 6.1 |
| 2019-W28 | 2018-19 | 39 | 1 | 4 | 26 | 3 | 5 | 1 | 4 | 6 | 2.6 | 10.3 | 66.7 | 7.7 | 12.8 | 2.6 | 10.3 | 15.4 |
| 2019-W29 | 2018-19 | 24 | 0 | 6 | 9 | 3 | 1 | 2 | 1 | 2 | 0.0 | 25.0 | 37.5 | 12.5 | 4.2 | 8.3 | 4.2 | 8.3 |
| 2019-W30 | 2018-19 | 34 | 0 | 4 | 11 | 1 | 0 | 1 | 3 | 2 | 0.0 | 11.8 | 32.4 | 2.9 | 0.0 | 2.9 | 8.8 | 5.9 |
| 2019-W31 | 2018-19 | 37 | 1 | 4 | 15 | 2 | 0 | 1 | 3 | 1 | 2.7 | 10.8 | 40.5 | 5.4 | 0.0 | 2.7 | 8.1 | 2.7 |
| 2019-W32 | 2018-19 | 42 | 0 | 3 | 13 | 3 | 2 | 0 | 0 | 2 | 0.0 | 7.1 | 31.0 | 7.1 | 4.8 | 0.0 | 0.0 | 4.8 |
| 2019-W33 | 2018-19 | 55 | 0 | 2 | 13 | 1 | 0 | 0 | 0 | 3 | 0.0 | 3.6 | 23.6 | 1.8 | 0.0 | 0.0 | 0.0 | 5.5 |
| 2019-W34 | 2018-19 | 92 | 0 | 4 | 6 | 0 | 1 | 1 | 1 | 1 | 0.0 | 4.3 | 6.5 | 0.0 | 1.1 | 1.1 | 1.1 | 1.1 |
| 2019-W35 | 2018-19 | 52 | 0 | 3 | 6 | 0 | 0 | 0 | 0 | 1 | 0.0 | 5.8 | 11.5 | 0.0 | 0.0 | 0.0 | 0.0 | 1.9 |
| 2019-W36 | 2019-20 | 41 | 0 | 1 | 3 | 1 | 0 | 0 | 0 | 0 | 0.0 | 2.4 | 7.3 | 2.4 | 0.0 | 0.0 | 0.0 | 0.0 |
| 2019-W37 | 2019-20 | 58 | 1 | 3 | 13 | 0 | 1 | 1 | 1 | 0 | 1.7 | 5.2 | 22.4 | 0.0 | 1.7 | 1.7 | 1.7 | 0.0 |
| 2019-W38 | 2019-20 | 76 | 0 | 3 | 14 | 4 | 1 | 1 | 1 | 0 | 0.0 | 3.9 | 18.4 | 5.3 | 1.3 | 1.3 | 1.3 | 0.0 |
| 2019-W39 | 2019-20 | 65 | 2 | 6 | 19 | 4 | 2 | 2 | 2 | 1 | 3.1 | 9.2 | 29.2 | 6.2 | 3.1 | 3.1 | 3.1 | 1.5 |
| 2019-W40 | 2019-20 | 67 | 1 | 4 | 29 | 2 | 1 | 0 | 2 | 1 | 1.5 | 6.0 | 43.3 | 3.0 | 1.5 | 0.0 | 3.0 | 1.5 |
| 2019-W41 | 2019-20 | 70 | 0 | 7 | 21 | 2 | 2 | 1 | 0 | 1 | 0.0 | 10.0 | 30.0 | 2.9 | 2.9 | 1.4 | 0.0 | 1.4 |
| 2019-W42 | 2019-20 | 69 | 1 | 3 | 23 | 4 | 0 | 2 | 2 | 7 | 1.4 | 4.3 | 33.3 | 5.8 | 0.0 | 2.9 | 2.9 | 10.1 |
| 2019-W43 | 2019-20 | 99 | 0 | 8 | 41 | 12 | 2 | 2 | 2 | 3 | 0.0 | 8.1 | 41.4 | 12.1 | 2.0 | 2.0 | 2.0 | 3.0 |
| 2019-W44 | 2019-20 | 98 | 1 | 6 | 29 | 9 | 3 | 3 | 1 | 2 | 1.0 | 6.1 | 29.6 | 9.2 | 3.1 | 3.1 | 1.0 | 2.0 |
| 2019-W45 | 2019-20 | 102 | 1 | 6 | 23 | 5 | 1 | 0 | 4 | 0 | 1.0 | 5.9 | 22.5 | 4.9 | 1.0 | 0.0 | 3.9 | 0.0 |
| 2019-W46 | 2019-20 | 90 | 1 | 14 | 23 | 4 | 1 | 3 | 1 | 3 | 1.1 | 15.6 | 25.6 | 4.4 | 1.1 | 3.3 | 1.1 | 3.3 |
| 2019-W47 | 2019-20 | 104 | 2 | 20 | 25 | 5 | 2 | 2 | 7 | 3 | 1.9 | 19.2 | 24.0 | 4.8 | 1.9 | 1.9 | 6.7 | 2.9 |
| 2019-W48 | 2019-20 | 108 | 2 | 26 | 36 | 1 | 0 | 8 | 1 | 2 | 1.9 | 24.1 | 33.3 | 0.9 | 0.0 | 7.4 | 0.9 | 1.9 |
| 2019-W49 | 2019-20 | 84 | 4 | 42 | 24 | 0 | 2 | 4 | 5 | 2 | 4.8 | 50.0 | 28.6 | 0.0 | 2.4 | 4.8 | 6.0 | 2.4 |
| 2019-W50 | 2019-20 | 132 | 2 | 45 | 26 | 4 | 5 | 6 | 4 | 2 | 1.5 | 34.1 | 19.7 | 3.0 | 3.8 | 4.5 | 3.0 | 1.5 |
| 2019-W51 | 2019-20 | 134 | 8 | 54 | 20 | 2 | 4 | 7 | 2 | 0 | 6.0 | 40.3 | 14.9 | 1.5 | 3.0 | 5.2 | 1.5 | 0.0 |
| 2019-W52 | 2019-20 | 121 | 11 | 50 | 24 | 3 | 4 | 7 | 3 | 4 | 9.1 | 41.3 | 19.8 | 2.5 | 3.3 | 5.8 | 2.5 | 3.3 |
| 2020-W01 | 2019-20 | 143 | 11 | 51 | 30 | 4 | 4 | 3 | 2 | 2 | 7.7 | 35.7 | 21.0 | 2.8 | 2.8 | 2.1 | 1.4 | 1.4 |
| 2020-W02 | 2019-20 | 123 | 4 | 40 | 20 | 1 | 3 | 8 | 5 | 4 | 3.3 | 32.5 | 16.3 | 0.8 | 2.4 | 6.5 | 4.1 | 3.3 |
| 2020-W03 | 2019-20 | 115 | 10 | 23 | 19 | 3 | 4 | 4 | 2 | 4 | 8.7 | 20.0 | 16.5 | 2.6 | 3.5 | 3.5 | 1.7 | 3.5 |
| 2020-W04 | 2019-20 | 112 | 7 | 15 | 28 | 3 | 2 | 6 | 2 | 5 | 6.2 | 13.4 | 25.0 | 2.7 | 1.8 | 5.4 | 1.8 | 4.5 |
| 2020-W05 | 2019-20 | 118 | 14 | 24 | 35 | 3 | 3 | 12 | 7 | 8 | 11.9 | 20.3 | 29.7 | 2.5 | 2.5 | 10.2 | 5.9 | 6.8 |
| 2020-W06 | 2019-20 | 120 | 24 | 13 | 46 | 7 | 6 | 16 | 4 | 6 | 20.0 | 10.8 | 38.3 | 5.8 | 5.0 | 13.3 | 3.3 | 5.0 |
| 2020-W07 | 2019-20 | 120 | 19 | 19 | 50 | 1 | 10 | 13 | 8 | 5 | 15.8 | 15.8 | 41.7 | 0.8 | 8.3 | 10.8 | 6.7 | 4.2 |
| 2020-W08 | 2019-20 | 113 | 23 | 21 | 55 | 2 | 12 | 12 | 7 | 3 | 20.4 | 18.6 | 48.7 | 1.8 | 10.6 | 10.6 | 6.2 | 2.7 |
| 2020-W09 | 2019-20 | 81 | 27 | 13 | 35 | 2 | 12 | 8 | 12 | 10 | 33.3 | 16.0 | 43.2 | 2.5 | 14.8 | 9.9 | 14.8 | 12.3 |
| 2020-W10 | 2019-20 | 75 | 11 | 11 | 30 | 2 | 9 | 4 | 3 | 6 | 14.7 | 14.7 | 40.0 | 2.7 | 12.0 | 5.3 | 4.0 | 8.0 |
| 2020-W11 | 2019-20 | 72 | 10 | 3 | 28 | 3 | 5 | 2 | 4 | 5 | 13.9 | 4.2 | 38.9 | 4.2 | 6.9 | 2.8 | 5.6 | 6.9 |
| 2020-W12 | 2019-20 | 108 | 6 | 11 | 28 | 2 | 7 | 5 | 4 | 3 | 5.6 | 10.2 | 25.9 | 1.9 | 6.5 | 4.6 | 3.7 | 2.8 |
| 2020-W13 | 2019-20 | 29 | 1 | 4 | 13 | 1 | 0 | 0 | 5 | 0 | 3.4 | 13.8 | 44.8 | 3.4 | 0.0 | 0.0 | 17.2 | 0.0 |
| 2020-W14 | 2019-20 | 29 | 0 | 2 | 8 | 1 | 2 | 0 | 4 | 1 | 0.0 | 6.9 | 27.6 | 3.4 | 6.9 | 0.0 | 13.8 | 3.4 |
| 2020-W15 | 2019-20 | 22 | 0 | 2 | 8 | 0 | 1 | 0 | 1 | 1 | 0.0 | 9.1 | 36.4 | 0.0 | 4.5 | 0.0 | 4.5 | 4.5 |
| 2020-W16 | 2019-20 | 26 | 2 | 1 | 8 | 0 | 0 | 0 | 1 | 2 | 7.7 | 3.8 | 30.8 | 0.0 | 0.0 | 0.0 | 3.8 | 7.7 |
| 2020-W17 | 2019-20 | 24 | 0 | 1 | 7 | 0 | 1 | 0 | 3 | 0 | 0.0 | 4.2 | 29.2 | 0.0 | 4.2 | 0.0 | 12.5 | 0.0 |
| 2020-W18 | 2019-20 | 29 | 0 | 2 | 7 | 0 | 0 | 0 | 0 | 1 | 0.0 | 6.9 | 24.1 | 0.0 | 0.0 | 0.0 | 0.0 | 3.4 |
| 2020-W19 | 2019-20 | 25 | 1 | 1 | 5 | 1 | 1 | 1 | 1 | 0 | 4.0 | 4.0 | 20.0 | 4.0 | 4.0 | 4.0 | 4.0 | 0.0 |
| 2020-W20 | 2019-20 | 35 | 0 | 1 | 7 | 0 | 1 | 1 | 0 | 2 | 0.0 | 2.9 | 20.0 | 0.0 | 2.9 | 2.9 | 0.0 | 5.7 |
| 2020-W21 | 2019-20 | 34 | 1 | 6 | 5 | 0 | 5 | 0 | 2 | 0 | 2.9 | 17.6 | 14.7 | 0.0 | 14.7 | 0.0 | 5.9 | 0.0 |
| 2020-W22 | 2019-20 | 33 | 0 | 1 | 5 | 0 | 4 | 0 | 2 | 2 | 0.0 | 3.0 | 15.2 | 0.0 | 12.1 | 0.0 | 6.1 | 6.1 |
| 2020-W23 | 2019-20 | 65 | 0 | 2 | 8 | 0 | 1 | 1 | 0 | 0 | 0.0 | 3.1 | 12.3 | 0.0 | 1.5 | 1.5 | 0.0 | 0.0 |
| 2020-W24 | 2019-20 | 28 | 0 | 1 | 2 | 0 | 2 | 0 | 1 | 0 | 0.0 | 3.6 | 7.1 | 0.0 | 7.1 | 0.0 | 3.6 | 0.0 |
| 2020-W25 | 2019-20 | 28 | 0 | 2 | 6 | 2 | 1 | 0 | 0 | 0 | 0.0 | 7.1 | 21.4 | 7.1 | 3.6 | 0.0 | 0.0 | 0.0 |
| 2020-W26 | 2019-20 | 14 | 0 | 2 | 4 | 1 | 0 | 0 | 2 | 1 | 0.0 | 14.3 | 28.6 | 7.1 | 0.0 | 0.0 | 14.3 | 7.1 |
| 2020-W27 | 2019-20 | 36 | 1 | 3 | 11 | 0 | 2 | 0 | 1 | 0 | 2.8 | 8.3 | 30.6 | 0.0 | 5.6 | 0.0 | 2.8 | 0.0 |
| 2020-W28 | 2019-20 | 31 | 0 | 4 | 18 | 1 | 0 | 0 | 2 | 0 | 0.0 | 12.9 | 58.1 | 3.2 | 0.0 | 0.0 | 6.5 | 0.0 |
| 2020-W29 | 2019-20 | 42 | 0 | 2 | 10 | 0 | 2 | 1 | 3 | 2 | 0.0 | 4.8 | 23.8 | 0.0 | 4.8 | 2.4 | 7.1 | 4.8 |
| 2020-W30 | 2019-20 | 42 | 1 | 2 | 22 | 3 | 2 | 0 | 0 | 1 | 2.4 | 4.8 | 52.4 | 7.1 | 4.8 | 0.0 | 0.0 | 2.4 |
| 2020-W31 | 2019-20 | 52 | 0 | 5 | 16 | 4 | 0 | 1 | 0 | 0 | 0.0 | 9.6 | 30.8 | 7.7 | 0.0 | 1.9 | 0.0 | 0.0 |
| 2020-W32 | 2019-20 | 58 | 0 | 4 | 13 | 1 | 2 | 1 | 0 | 1 | 0.0 | 6.9 | 22.4 | 1.7 | 3.4 | 1.7 | 0.0 | 1.7 |
| 2020-W33 | 2019-20 | 57 | 1 | 8 | 13 | 0 | 3 | 0 | 1 | 0 | 1.8 | 14.0 | 22.8 | 0.0 | 5.3 | 0.0 | 1.8 | 0.0 |
| 2020-W34 | 2019-20 | 75 | 0 | 5 | 9 | 0 | 2 | 1 | 1 | 0 | 0.0 | 6.7 | 12.0 | 0.0 | 2.7 | 1.3 | 1.3 | 0.0 |
| 2020-W35 | 2019-20 | 48 | 1 | 4 | 16 | 1 | 2 | 2 | 1 | 2 | 2.1 | 8.3 | 33.3 | 2.1 | 4.2 | 4.2 | 2.1 | 4.2 |
| 2020-W36 | 2020-21 | 31 | 0 | 2 | 8 | 1 | 0 | 2 | 2 | 2 | 0.0 | 6.5 | 25.8 | 3.2 | 0.0 | 6.5 | 6.5 | 6.5 |
| 2020-W37 | 2020-21 | 77 | 5 | 8 | 38 | 1 | 1 | 4 | 2 | 0 | 6.5 | 10.4 | 49.4 | 1.3 | 1.3 | 5.2 | 2.6 | 0.0 |
| 2020-W38 | 2020-21 | 87 | 3 | 6 | 35 | 0 | 3 | 2 | 1 | 2 | 3.4 | 6.9 | 40.2 | 0.0 | 3.4 | 2.3 | 1.1 | 2.3 |
| 2020-W39 | 2020-21 | 66 | 1 | 9 | 33 | 0 | 1 | 1 | 0 | 4 | 1.5 | 13.6 | 50.0 | 0.0 | 1.5 | 1.5 | 0.0 | 6.1 |
| 2020-W40 | 2020-21 | 64 | 1 | 4 | 21 | 2 | 1 | 1 | 3 | 2 | 1.6 | 6.2 | 32.8 | 3.1 | 1.6 | 1.6 | 4.7 | 3.1 |
| 2020-W41 | 2020-21 | 134 | 1 | 5 | 17 | 1 | 3 | 0 | 3 | 3 | 0.7 | 3.7 | 12.7 | 0.7 | 2.2 | 0.0 | 2.2 | 2.2 |
| 2020-W42 | 2020-21 | 145 | 3 | 6 | 29 | 0 | 0 | 2 | 4 | 3 | 2.1 | 4.1 | 20.0 | 0.0 | 0.0 | 1.4 | 2.8 | 2.1 |
| 2020-W43 | 2020-21 | 137 | 2 | 3 | 32 | 1 | 2 | 2 | 3 | 5 | 1.5 | 2.2 | 23.4 | 0.7 | 1.5 | 1.5 | 2.2 | 3.6 |
| 2020-W44 | 2020-21 | 114 | 1 | 2 | 25 | 0 | 3 | 2 | 3 | 2 | 0.9 | 1.8 | 21.9 | 0.0 | 2.6 | 1.8 | 2.6 | 1.8 |
| 2020-W45 | 2020-21 | 96 | 1 | 6 | 21 | 0 | 0 | 2 | 4 | 1 | 1.0 | 6.2 | 21.9 | 0.0 | 0.0 | 2.1 | 4.2 | 1.0 |
| 2020-W46 | 2020-21 | 172 | 2 | 3 | 41 | 2 | 1 | 3 | 3 | 14 | 1.2 | 1.7 | 23.8 | 1.2 | 0.6 | 1.7 | 1.7 | 8.1 |
| 2020-W47 | 2020-21 | 116 | 0 | 1 | 50 | 0 | 1 | 1 | 1 | 14 | 0.0 | 0.9 | 43.1 | 0.0 | 0.9 | 0.9 | 0.9 | 12.1 |
| 2020-W48 | 2020-21 | 133 | 2 | 6 | 51 | 0 | 2 | 2 | 1 | 13 | 1.5 | 4.5 | 38.3 | 0.0 | 1.5 | 1.5 | 0.8 | 9.8 |
| 2020-W49 | 2020-21 | 125 | 1 | 5 | 38 | 2 | 3 | 4 | 1 | 12 | 0.8 | 4.0 | 30.4 | 1.6 | 2.4 | 3.2 | 0.8 | 9.6 |
| 2020-W50 | 2020-21 | 102 | 0 | 4 | 63 | 3 | 2 | 3 | 1 | 19 | 0.0 | 3.9 | 61.8 | 2.9 | 2.0 | 2.9 | 1.0 | 18.6 |
| 2020-W51 | 2020-21 | 106 | 1 | 6 | 47 | 2 | 0 | 6 | 2 | 15 | 0.9 | 5.7 | 44.3 | 1.9 | 0.0 | 5.7 | 1.9 | 14.2 |
| 2020-W52 | 2020-21 | 101 | 0 | 2 | 47 | 8 | 1 | 1 | 4 | 8 | 0.0 | 2.0 | 46.5 | 7.9 | 1.0 | 1.0 | 4.0 | 7.9 |
| 2020-W53 | 2020-21 | 108 | 0 | 9 | 48 | 7 | 5 | 5 | 12 | 10 | 0.0 | 8.3 | 44.4 | 6.5 | 4.6 | 4.6 | 11.1 | 9.3 |
| 2021-W01 | 2020-21 | 107 | 0 | 8 | 29 | 12 | 1 | 3 | 6 | 9 | 0.0 | 7.5 | 27.1 | 11.2 | 0.9 | 2.8 | 5.6 | 8.4 |
| 2021-W02 | 2020-21 | 136 | 1 | 4 | 39 | 13 | 2 | 7 | 10 | 7 | 0.7 | 2.9 | 28.7 | 9.6 | 1.5 | 5.1 | 7.4 | 5.1 |
| 2021-W03 | 2020-21 | 132 | 0 | 5 | 49 | 2 | 2 | 4 | 5 | 13 | 0.0 | 3.8 | 37.1 | 1.5 | 1.5 | 3.0 | 3.8 | 9.8 |
| 2021-W04 | 2020-21 | 178 | 1 | 8 | 39 | 28 | 3 | 7 | 10 | 5 | 0.6 | 4.5 | 21.9 | 15.7 | 1.7 | 3.9 | 5.6 | 2.8 |
| 2021-W05 | 2020-21 | 207 | 0 | 10 | 51 | 33 | 4 | 5 | 10 | 6 | 0.0 | 4.8 | 24.6 | 15.9 | 1.9 | 2.4 | 4.8 | 2.9 |
| 2021-W06 | 2020-21 | 228 | 1 | 20 | 43 | 29 | 6 | 7 | 19 | 9 | 0.4 | 8.8 | 18.9 | 12.7 | 2.6 | 3.1 | 8.3 | 3.9 |
| 2021-W07 | 2020-21 | 187 | 1 | 25 | 46 | 31 | 8 | 10 | 12 | 6 | 0.5 | 13.4 | 24.6 | 16.6 | 4.3 | 5.3 | 6.4 | 3.2 |
| 2021-W08 | 2020-21 | 124 | 1 | 30 | 47 | 25 | 11 | 14 | 19 | 12 | 0.8 | 24.2 | 37.9 | 20.2 | 8.9 | 11.3 | 15.3 | 9.7 |
| 2021-W09 | 2020-21 | 91 | 0 | 35 | 29 | 16 | 18 | 13 | 10 | 12 | 0.0 | 38.5 | 31.9 | 17.6 | 19.8 | 14.3 | 11.0 | 13.2 |
| 2021-W10 | 2020-21 | 105 | 1 | 25 | 30 | 9 | 12 | 12 | 20 | 8 | 1.0 | 23.8 | 28.6 | 8.6 | 11.4 | 11.4 | 19.0 | 7.6 |
| 2021-W11 | 2020-21 | 91 | 1 | 32 | 29 | 6 | 14 | 11 | 8 | 6 | 1.1 | 35.2 | 31.9 | 6.6 | 15.4 | 12.1 | 8.8 | 6.6 |
| 2021-W12 | 2020-21 | 157 | 0 | 45 | 64 | 15 | 24 | 11 | 15 | 11 | 0.0 | 28.7 | 40.8 | 9.6 | 15.3 | 7.0 | 9.6 | 7.0 |
| 2021-W13 | 2020-21 | 110 | 2 | 48 | 58 | 4 | 40 | 16 | 10 | 7 | 1.8 | 43.6 | 52.7 | 3.6 | 36.4 | 14.5 | 9.1 | 6.4 |
| 2021-W14 | 2020-21 | 96 | 2 | 55 | 65 | 7 | 38 | 19 | 17 | 11 | 2.1 | 57.3 | 67.7 | 7.3 | 39.6 | 19.8 | 17.7 | 11.5 |
| 2021-W15 | 2020-21 | 122 | 5 | 39 | 38 | 3 | 18 | 12 | 16 | 16 | 4.1 | 32.0 | 31.1 | 2.5 | 14.8 | 9.8 | 13.1 | 13.1 |
| 2021-W16 | 2020-21 | 96 | 0 | 26 | 28 | 2 | 11 | 6 | 8 | 4 | 0.0 | 27.1 | 29.2 | 2.1 | 11.5 | 6.2 | 8.3 | 4.2 |
| 2021-W17 | 2020-21 | 113 | 0 | 13 | 13 | 4 | 8 | 3 | 4 | 4 | 0.0 | 11.5 | 11.5 | 3.5 | 7.1 | 2.7 | 3.5 | 3.5 |
| 2021-W18 | 2020-21 | 132 | 1 | 15 | 22 | 4 | 9 | 3 | 6 | 6 | 0.8 | 11.4 | 16.7 | 3.0 | 6.8 | 2.3 | 4.5 | 4.5 |
| 2021-W19 | 2020-21 | 125 | 1 | 11 | 25 | 4 | 8 | 1 | 7 | 5 | 0.8 | 8.8 | 20.0 | 3.2 | 6.4 | 0.8 | 5.6 | 4.0 |
| 2021-W20 | 2020-21 | 135 | 2 | 13 | 34 | 3 | 2 | 4 | 3 | 4 | 1.5 | 9.6 | 25.2 | 2.2 | 1.5 | 3.0 | 2.2 | 3.0 |
| 2021-W21 | 2020-21 | 104 | 0 | 14 | 27 | 5 | 3 | 1 | 1 | 3 | 0.0 | 13.5 | 26.0 | 4.8 | 2.9 | 1.0 | 1.0 | 2.9 |
| 2021-W22 | 2020-21 | 144 | 1 | 11 | 43 | 7 | 5 | 3 | 3 | 5 | 0.7 | 7.6 | 29.9 | 4.9 | 3.5 | 2.1 | 2.1 | 3.5 |
| 2021-W23 | 2020-21 | 159 | 1 | 14 | 39 | 5 | 6 | 2 | 0 | 4 | 0.6 | 8.8 | 24.5 | 3.1 | 3.8 | 1.3 | 0.0 | 2.5 |
| 2021-W24 | 2020-21 | 138 | 0 | 25 | 50 | 2 | 2 | 1 | 3 | 2 | 0.0 | 18.1 | 36.2 | 1.4 | 1.4 | 0.7 | 2.2 | 1.4 |
| 2021-W25 | 2020-21 | 124 | 1 | 21 | 53 | 8 | 0 | 4 | 8 | 5 | 0.8 | 16.9 | 42.7 | 6.5 | 0.0 | 3.2 | 6.5 | 4.0 |
| 2021-W26 | 2020-21 | 111 | 5 | 23 | 46 | 14 | 3 | 7 | 4 | 6 | 4.5 | 20.7 | 41.4 | 12.6 | 2.7 | 6.3 | 3.6 | 5.4 |
| 2021-W27 | 2020-21 | 100 | 1 | 34 | 53 | 17 | 3 | 7 | 2 | 11 | 1.0 | 34.0 | 53.0 | 17.0 | 3.0 | 7.0 | 2.0 | 11.0 |
| 2021-W28 | 2020-21 | 100 | 3 | 30 | 41 | 8 | 2 | 4 | 10 | 8 | 3.0 | 30.0 | 41.0 | 8.0 | 2.0 | 4.0 | 10.0 | 8.0 |
| 2021-W29 | 2020-21 | 109 | 1 | 36 | 36 | 17 | 0 | 4 | 6 | 6 | 0.9 | 33.0 | 33.0 | 15.6 | 0.0 | 3.7 | 5.5 | 5.5 |
| 2021-W30 | 2020-21 | 164 | 3 | 26 | 51 | 14 | 2 | 2 | 5 | 7 | 1.8 | 15.9 | 31.1 | 8.5 | 1.2 | 1.2 | 3.0 | 4.3 |
| 2021-W31 | 2020-21 | 160 | 0 | 21 | 48 | 16 | 2 | 3 | 4 | 2 | 0.0 | 13.1 | 30.0 | 10.0 | 1.2 | 1.9 | 2.5 | 1.2 |
| 2021-W32 | 2020-21 | 168 | 1 | 11 | 19 | 8 | 2 | 2 | 2 | 7 | 0.6 | 6.5 | 11.3 | 4.8 | 1.2 | 1.2 | 1.2 | 4.2 |
| 2021-W33 | 2020-21 | 159 | 2 | 12 | 20 | 9 | 1 | 4 | 3 | 3 | 1.3 | 7.5 | 12.6 | 5.7 | 0.6 | 2.5 | 1.9 | 1.9 |
| 2021-W34 | 2020-21 | 159 | 2 | 8 | 21 | 10 | 1 | 4 | 2 | 2 | 1.3 | 5.0 | 13.2 | 6.3 | 0.6 | 2.5 | 1.3 | 1.3 |
| 2021-W35 | 2020-21 | 128 | 0 | 7 | 21 | 6 | 0 | 0 | 3 | 3 | 0.0 | 5.5 | 16.4 | 4.7 | 0.0 | 0.0 | 2.3 | 2.3 |
| 2021-W36 | 2021-22 | 131 | 0 | 5 | 36 | 4 | 1 | 1 | 2 | 3 | 0.0 | 3.8 | 27.5 | 3.1 | 0.8 | 0.8 | 1.5 | 2.3 |
| 2021-W37 | 2021-22 | 120 | 0 | 5 | 55 | 1 | 3 | 0 | 3 | 2 | 0.0 | 4.2 | 45.8 | 0.8 | 2.5 | 0.0 | 2.5 | 1.7 |
| 2021-W38 | 2021-22 | 147 | 0 | 12 | 87 | 3 | 1 | 2 | 2 | 2 | 0.0 | 8.2 | 59.2 | 2.0 | 0.7 | 1.4 | 1.4 | 1.4 |
| 2021-W39 | 2021-22 | 153 | 0 | 19 | 102 | 5 | 3 | 1 | 2 | 8 | 0.0 | 12.4 | 66.7 | 3.3 | 2.0 | 0.7 | 1.3 | 5.2 |
| 2021-W40 | 2021-22 | 198 | 0 | 35 | 106 | 8 | 2 | 5 | 7 | 6 | 0.0 | 17.7 | 53.5 | 4.0 | 1.0 | 2.5 | 3.5 | 3.0 |
| 2021-W41 | 2021-22 | 169 | 0 | 34 | 91 | 9 | 3 | 5 | 3 | 8 | 0.0 | 20.1 | 53.8 | 5.3 | 1.8 | 3.0 | 1.8 | 4.7 |
| 2021-W42 | 2021-22 | 222 | 0 | 65 | 91 | 9 | 4 | 10 | 8 | 11 | 0.0 | 29.3 | 41.0 | 4.1 | 1.8 | 4.5 | 3.6 | 5.0 |
| 2021-W43 | 2021-22 | 215 | 0 | 74 | 97 | 10 | 8 | 6 | 21 | 16 | 0.0 | 34.4 | 45.1 | 4.7 | 3.7 | 2.8 | 9.8 | 7.4 |
| 2021-W44 | 2021-22 | 177 | 0 | 75 | 56 | 14 | 3 | 4 | 5 | 8 | 0.0 | 42.4 | 31.6 | 7.9 | 1.7 | 2.3 | 2.8 | 4.5 |
| 2021-W45 | 2021-22 | 163 | 0 | 60 | 27 | 4 | 2 | 13 | 6 | 7 | 0.0 | 36.8 | 16.6 | 2.5 | 1.2 | 8.0 | 3.7 | 4.3 |
| 2021-W46 | 2021-22 | 149 | 2 | 30 | 29 | 4 | 6 | 8 | 4 | 7 | 1.3 | 20.1 | 19.5 | 2.7 | 4.0 | 5.4 | 2.7 | 4.7 |
| 2021-W47 | 2021-22 | 214 | 6 | 59 | 53 | 4 | 3 | 12 | 4 | 2 | 2.8 | 27.6 | 24.8 | 1.9 | 1.4 | 5.6 | 1.9 | 0.9 |
| 2021-W48 | 2021-22 | 222 | 5 | 57 | 47 | 6 | 5 | 25 | 3 | 13 | 2.3 | 25.7 | 21.2 | 2.7 | 2.3 | 11.3 | 1.4 | 5.9 |
| 2021-W49 | 2021-22 | 228 | 7 | 52 | 39 | 6 | 2 | 18 | 3 | 8 | 3.1 | 22.8 | 17.1 | 2.6 | 0.9 | 7.9 | 1.3 | 3.5 |
| 2021-W50 | 2021-22 | 221 | 10 | 46 | 47 | 8 | 8 | 17 | 4 | 13 | 4.5 | 20.8 | 21.3 | 3.6 | 3.6 | 7.7 | 1.8 | 5.9 |
| 2021-W51 | 2021-22 | 233 | 14 | 28 | 48 | 16 | 16 | 31 | 11 | 8 | 6.0 | 12.0 | 20.6 | 6.9 | 6.9 | 13.3 | 4.7 | 3.4 |
| 2021-W52 | 2021-22 | 267 | 18 | 32 | 49 | 22 | 17 | 20 | 15 | 17 | 6.7 | 12.0 | 18.4 | 8.2 | 6.4 | 7.5 | 5.6 | 6.4 |
| 2022-W01 | 2021-22 | 542 | 11 | 35 | 39 | 8 | 26 | 11 | 13 | 13 | 2.0 | 6.5 | 7.2 | 1.5 | 4.8 | 2.0 | 2.4 | 2.4 |
| 2022-W02 | 2021-22 | 758 | 27 | 24 | 33 | 3 | 10 | 8 | 6 | 13 | 3.6 | 3.2 | 4.4 | 0.4 | 1.3 | 1.1 | 0.8 | 1.7 |
| 2022-W03 | 2021-22 | 703 | 23 | 15 | 43 | 15 | 19 | 13 | 5 | 6 | 3.3 | 2.1 | 6.1 | 2.1 | 2.7 | 1.8 | 0.7 | 0.9 |
| 2022-W04 | 2021-22 | 661 | 36 | 13 | 41 | 2 | 9 | 2 | 11 | 13 | 5.4 | 2.0 | 6.2 | 0.3 | 1.4 | 0.3 | 1.7 | 2.0 |
| 2022-W05 | 2021-22 | 668 | 39 | 14 | 57 | 7 | 20 | 3 | 26 | 15 | 5.8 | 2.1 | 8.5 | 1.0 | 3.0 | 0.4 | 3.9 | 2.2 |
| 2022-W06 | 2021-22 | 480 | 43 | 18 | 50 | 6 | 15 | 5 | 13 | 15 | 9.0 | 3.8 | 10.4 | 1.2 | 3.1 | 1.0 | 2.7 | 3.1 |
| 2022-W07 | 2021-22 | 383 | 29 | 8 | 48 | 4 | 13 | 4 | 7 | 11 | 7.6 | 2.1 | 12.5 | 1.0 | 3.4 | 1.0 | 1.8 | 2.9 |
| 2022-W08 | 2021-22 | 341 | 48 | 2 | 37 | 3 | 6 | 2 | 7 | 11 | 14.1 | 0.6 | 10.9 | 0.9 | 1.8 | 0.6 | 2.1 | 3.2 |
| 2022-W09 | 2021-22 | 401 | 81 | 3 | 52 | 2 | 6 | 3 | 10 | 13 | 20.2 | 0.7 | 13.0 | 0.5 | 1.5 | 0.7 | 2.5 | 3.2 |
| 2022-W10 | 2021-22 | 514 | 143 | 5 | 69 | 1 | 7 | 2 | 15 | 8 | 27.8 | 1.0 | 13.4 | 0.2 | 1.4 | 0.4 | 2.9 | 1.6 |
| 2022-W11 | 2021-22 | 613 | 149 | 6 | 92 | 5 | 11 | 2 | 11 | 21 | 24.3 | 1.0 | 15.0 | 0.8 | 1.8 | 0.3 | 1.8 | 3.4 |
| 2022-W12 | 2021-22 | 594 | 137 | 3 | 85 | 4 | 7 | 5 | 15 | 23 | 23.1 | 0.5 | 14.3 | 0.7 | 1.2 | 0.8 | 2.5 | 3.9 |
| 2022-W13 | 2021-22 | 623 | 188 | 4 | 82 | 7 | 7 | 1 | 11 | 16 | 30.2 | 0.6 | 13.2 | 1.1 | 1.1 | 0.2 | 1.8 | 2.6 |
| 2022-W14 | 2021-22 | 469 | 118 | 3 | 49 | 7 | 14 | 1 | 9 | 21 | 25.2 | 0.6 | 10.4 | 1.5 | 3.0 | 0.2 | 1.9 | 4.5 |
| 2022-W15 | 2021-22 | 427 | 127 | 1 | 54 | 4 | 6 | 3 | 4 | 14 | 29.7 | 0.2 | 12.6 | 0.9 | 1.4 | 0.7 | 0.9 | 3.3 |
| 2022-W16 | 2021-22 | 324 | 42 | 2 | 47 | 6 | 8 | 1 | 1 | 11 | 13.0 | 0.6 | 14.5 | 1.9 | 2.5 | 0.3 | 0.3 | 3.4 |
| 2022-W17 | 2021-22 | 314 | 14 | 1 | 39 | 6 | 3 | 0 | 5 | 10 | 4.5 | 0.3 | 12.4 | 1.9 | 1.0 | 0.0 | 1.6 | 3.2 |
| 2022-W18 | 2021-22 | 246 | 13 | 0 | 22 | 1 | 1 | 1 | 3 | 8 | 5.3 | 0.0 | 8.9 | 0.4 | 0.4 | 0.4 | 1.2 | 3.3 |
| 2022-W19 | 2021-22 | 269 | 8 | 2 | 51 | 9 | 3 | 2 | 3 | 13 | 3.0 | 0.7 | 19.0 | 3.3 | 1.1 | 0.7 | 1.1 | 4.8 |
| 2022-W20 | 2021-22 | 299 | 4 | 1 | 60 | 7 | 2 | 2 | 4 | 4 | 1.3 | 0.3 | 20.1 | 2.3 | 0.7 | 0.7 | 1.3 | 1.3 |
| 2022-W21 | 2021-22 | 254 | 4 | 2 | 54 | 14 | 6 | 4 | 1 | 9 | 1.6 | 0.8 | 21.3 | 5.5 | 2.4 | 1.6 | 0.4 | 3.5 |
| 2022-W22 | 2021-22 | 219 | 1 | 2 | 63 | 22 | 1 | 0 | 0 | 13 | 0.5 | 0.9 | 28.8 | 10.0 | 0.5 | 0.0 | 0.0 | 5.9 |
| 2022-W23 | 2021-22 | 218 | 2 | 2 | 59 | 20 | 1 | 5 | 5 | 10 | 0.9 | 0.9 | 27.1 | 9.2 | 0.5 | 2.3 | 2.3 | 4.6 |
| 2022-W24 | 2021-22 | 208 | 0 | 4 | 59 | 15 | 3 | 3 | 5 | 7 | 0.0 | 1.9 | 28.4 | 7.2 | 1.4 | 1.4 | 2.4 | 3.4 |
| 2022-W25 | 2021-22 | 235 | 1 | 0 | 64 | 25 | 3 | 11 | 3 | 14 | 0.4 | 0.0 | 27.2 | 10.6 | 1.3 | 4.7 | 1.3 | 6.0 |
| 2022-W26 | 2021-22 | 232 | 1 | 2 | 52 | 19 | 3 | 7 | 2 | 21 | 0.4 | 0.9 | 22.4 | 8.2 | 1.3 | 3.0 | 0.9 | 9.1 |
| 2022-W27 | 2021-22 | 275 | 0 | 2 | 57 | 18 | 1 | 9 | 1 | 15 | 0.0 | 0.7 | 20.7 | 6.5 | 0.4 | 3.3 | 0.4 | 5.5 |
| 2022-W28 | 2021-22 | 274 | 0 | 3 | 56 | 14 | 3 | 3 | 4 | 10 | 0.0 | 1.1 | 20.4 | 5.1 | 1.1 | 1.1 | 1.5 | 3.6 |
| 2022-W29 | 2021-22 | 231 | 2 | 7 | 34 | 8 | 1 | 1 | 2 | 14 | 0.9 | 3.0 | 14.7 | 3.5 | 0.4 | 0.4 | 0.9 | 6.1 |
| 2022-W30 | 2021-22 | 207 | 0 | 4 | 27 | 12 | 4 | 4 | 4 | 15 | 0.0 | 1.9 | 13.0 | 5.8 | 1.9 | 1.9 | 1.9 | 7.2 |
| 2022-W31 | 2021-22 | 170 | 0 | 3 | 18 | 12 | 0 | 7 | 1 | 12 | 0.0 | 1.8 | 10.6 | 7.1 | 0.0 | 4.1 | 0.6 | 7.1 |
| 2022-W32 | 2021-22 | 179 | 1 | 4 | 11 | 7 | 0 | 2 | 1 | 9 | 0.6 | 2.2 | 6.1 | 3.9 | 0.0 | 1.1 | 0.6 | 5.0 |
| 2022-W33 | 2021-22 | 157 | 0 | 2 | 14 | 4 | 2 | 3 | 1 | 10 | 0.0 | 1.3 | 8.9 | 2.5 | 1.3 | 1.9 | 0.6 | 6.4 |
| 2022-W34 | 2021-22 | 148 | 1 | 2 | 13 | 4 | 1 | 1 | 2 | 6 | 0.7 | 1.4 | 8.8 | 2.7 | 0.7 | 0.7 | 1.4 | 4.1 |
| 2022-W35 | 2021-22 | 85 | 0 | 1 | 4 | 0 | 1 | 0 | 0 | 6 | 0.0 | 1.2 | 4.7 | 0.0 | 1.2 | 0.0 | 0.0 | 7.1 |
