# Supplementary material for: Characteristics of respiratory viruses’ circulation through a six-year period (2016–2022) in a pediatric population in Normandy, France, and the impact of COVID-19 pandemic
Source: Microbiol Spectr. 2023 Oct 26;11(6):e01867-23. doi: 10.1128/spectrum.01867-23 (PMC10714951; doi:10.1128/spectrum.01867-23)
Supplement: Table S2 — Assays used and viruses detected by each test. [file spectrum.01867-23-s0004.docx]

**Supplementary Table 2. Assays used and viruses detected**

| Test | Viral Pathogens detected |
| --- | --- |
| NxTAG Respiratory Pathogen Panel (Luminex, Austin, TX, USA) | Influenza A, AH1,AH3,B; RSVA,RSVB, Rh/EV, PIV1,2,3,4, CoV HKU1, NL63, 229E, OC43, HBoV |
| ePlex® Respiratory Pathogen (Genmark Diagnopstics, Inc. California, USA)  and ePlex®RP2 (version2) | Influenza A, AH1,AH1-pdm09, AH3, B; RSVA,RSVB, Rh/EV, PIV1,2,3,4, CoV HKU1, NL63, 229E, OC43, HBoV and SARS-CoV-2 in RP2 |
| Allplex™ Respiratory panel (Seegene, Seoul, South Korea), | Influenza A, AH1,AH1-pdm09, AH3, B; RSVA,RSVB and SARS-CoV-2 |
| Panther Fusion Flu A/B/RSV and AdV/hMPV/RV assays (Hologic, Inc., San Diego, CA, USA) | Influenza A/B, RSV, AdV, HMPV/RV |
| Sofia® Influenza and RSV | Influenza A,B and RSV |
| Cobas® Liat® A testing /B & RSV | Influenza A,B and RSV |
| Xpert Flu/RSV and Xpert SARS-CoV-2/Flu/RSV tests (Cepheid, Inc., Sunnyvale, CA) | SARS-CoV2, InfluenzaA,B and RSV |
| ViroQ^®^ SARS-FluA/B-RSV kit (BAG Diagnostics, Lich, Germany) | SARS-CoV2, InfluenzaA,B and RSV |
